# Non-Contact State Assessment of Falling-Film Flow over Horizontal Tube Bundles Using High-Speed Imaging

**DOI:** 10.3390/s26134073

**Published:** 2026-06-26

**Authors:** Weida Wang, Maocheng Tian, Guanmin Zhang, Yan Qiu

**Affiliations:** 1 School of Nuclear Science, Energy and Power Engineering, Shandong University, Jinan 250061, China; 201920473@mail.sdu.edu.cn (W.W.); zhgm@sdu.edu.cn (G.Z.); anneqiu@sdu.edu.cn (Y.Q.); 2 Shandong Key Laboratory of Thermal Science and Smart Energy Systems, Jinan 250061, China

**Keywords:** high-speed imaging, visual-proxy sensing, falling-film flow, horizontal tube bundle, non-contact monitoring

## Abstract

**Highlights:**

**What are the main findings?**

- A visual-proxy sensing framework was developed to convert high-speed reflective images of falling-film flow over horizontal tube bundles into normalized spatiotemporal maps, dynamic renewal fields, and interpretable image-derived descriptors.
- The extracted descriptors identify the transition from sparse column flow to more continuous sheet flow and reveal row-dependent differences in renewal intensity, high-frequency optical activity, characteristic time scale, and threshold-defined burst-like events.

**What are the implication of the main findings?**

- Zero-response, noise-correction, and parameter-sensitivity tests support the robustness of the method for low-calibration, non-contact comparative monitoring.
- The descriptors provide relative image-based optical proxies for diagnostic comparison and should not be interpreted as absolute film-thickness or velocity measurements.

**Abstract:**

High-speed imaging offers a non-intrusive approach for monitoring falling-film flows over horizontal tube bundles, but reflective images are difficult to quantify because grayscale variations are jointly affected by film geometry, interfacial curvature, surface slope, viewing angle, and local highlights. This study proposes an interpretable visual-proxy sensing framework for comparative state assessment of such flows. Isothermal water experiments were conducted on a five-row horizontal tube bundle over $Re_{\Gamma }=184–960$. For each condition, 2000 grayscale frames were acquired at 2000 fps and analyzed within five fixed row-wise regions of interest. The image sequence was transformed by temporal-median background subtraction, local spatiotemporal mapping, moving-average detrending, and median-absolute-deviation normalization. The resulting normalized map $M_{n}$ and dynamic renewal field G were used to extract four scalar descriptors: noise-corrected apparent renewal intensity $I_{R}$, high-frequency fraction $R_{HF}$, spectral peak frequency $f_{p}$, and burst-event rate $F_{B}$. Results show that $M_{n}$ and G capture the transition from sparse column flow to more continuous sheet flow and reveal row-dependent activity organization. The descriptors provide complementary information on renewal intensity, frequency composition, dominant time scale, and intermittent events. Zero-response, noise-correction, and sensitivity tests confirm that the framework avoids structured pseudo-waves and maintains stable row-wise comparisons. The method provides a low-calibration visual sensing tool for relative falling-film state assessment.

## 1. Introduction

**Figure 1:**
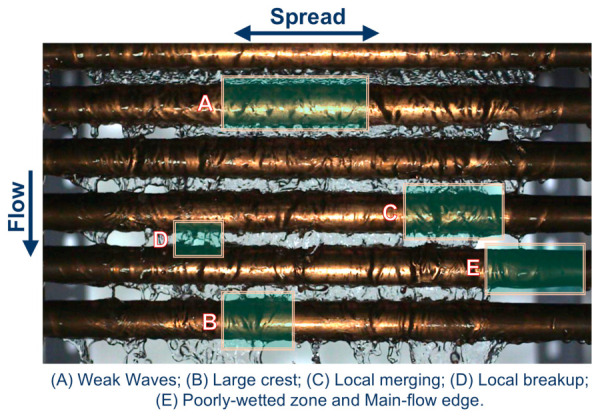
Typical visual features of falling-film flow over a horizontal tube bundle. The arrows indicate the streamwise falling direction and the axial spreading direction of the liquid film. The annotated regions illustrate representative reflective-image features: (A) weak waves, (B) large crest, (C) local merging of liquid structures, (D) local breakup, and (E) poorly wetted zone and main-flow edge.

Falling-film flow over horizontal tube bundles is widely encountered in industrial applications such as refrigeration, chemical engineering, petroleum refining, and seawater desalination, particularly in equipment such as multi-effect distillation units and falling-film evaporators [1]. Figure 1 shows typical visualized characteristics of falling film flow. Driven by gravity, the liquid film undergoes free fall and impingement between adjacent tube rows, wets and spreads over the tube surface, and develops interfacial ripples along the tube wall, producing distinct light–dark variations and non-uniform structures. The spatial distribution of the liquid film and the interfacial flow behavior directly affect the operating efficiency and stability of these devices, and intensified local fluctuations can indicate the onset of performance degradation [2,3]. Therefore, rapid identification of the flow regime [4], interfacial activity level [5], and instability tendency of the falling film is important for evaluating equipment performance and monitoring operating conditions [6]. High-speed visualization-based image sensing methods provide a promising diagnostic approach because of their non-invasive nature, high temporal resolution, and large field of view.

Table 1 summarizes the main existing techniques for measuring liquid-film interfaces. Contact probes can provide only local time series of film thickness and inevitably perturb the film, making it difficult to capture full-field spreading and propagation characteristics [7,8,9,10]. Non-contact optical techniques such as planar laser-induced fluorescence and confocal chromatic sensing can reconstruct film-thickness distributions or velocity-related information in suitable optical configurations, with reasonable accuracy under controlled conditions [7,11,12]. However, for unsteady flow over curved surfaces in multi-row tube bundles, where refraction, reflection, and interface deformation are complex, these methods typically require careful optical alignment and calibration, and some require dyes or tracers in the working fluid [13,14,15,16], which limits their deployment in engineering-oriented scenarios. In contrast, high-speed visualization using conventional light sources can provide continuous image sequences of the spatiotemporal flow evolution at relatively low system complexity [17,18], without perturbing the film. Nevertheless, raw high-speed images mainly provide qualitative flow information. For quantitative processing, they are affected by local curvature, interfacial slope, light refraction, and dynamic shadows [13,19], and therefore cannot be directly converted into absolute physical quantities [20].

It is therefore necessary to analyze and compare image sequences using robust image-processing methods, so as to identify the falling-film flow regime and evaluate relative differences in flow behavior. Under fixed imaging conditions, grayscale fluctuations in high-speed reflective images can contain information on relative dynamic features, such as interfacial activity, wave propagation, and local burst-like events, and can be processed using signal-processing techniques based on robust statistics and frequency-domain analysis [21]. Existing methods, including optical flow [22], STIV/kymograph analysis [23], spatiotemporal cross-correlation [24], frequency–wavenumber spectrum analysis [25], and POD/DMD modal decomposition [26], can extract motion or structural information from image sequences without contacting the flow field. Their suitability depends on the diagnostic objective and the assumptions made about image formation. Optical flow, STIV, and cross-correlation are well suited to displacement- or velocity-oriented analyses when trackable textures and sufficient brightness consistency are available. Frequency–wavenumber analysis can identify propagating spectral components under adequate spatial and temporal sampling, and POD/DMD can reveal dominant coherent structures and reduce data dimensionality. In the reflective falling-film images considered in this study, grayscale patterns are shaped by coupled effects of material motion, film-thickness variation, interfacial curvature, surface slope, wetting-boundary migration, local highlights, and illumination response. These image-formation characteristics motivate a descriptor-oriented optical-proxy framework for comparative state assessment under fixed imaging conditions.

For high-speed reflective images of falling-film flow over tube bundles, local grayscale variations do not correspond strictly to material-point translation or absolute changes in film thickness; instead, they are jointly affected by interfacial fluctuations, curvature, reflection angle, and local highlight renewal. Therefore, velocity estimation, streak inclination, or modal decomposition alone is insufficient to fully describe the local interfacial activity [27]. On this basis, this study develops a proxy-descriptor framework for high-speed reflective images. Through robust background modeling, intensity normalization, temporal-gradient extraction, spectral decomposition, event identification, and kymograph-based propagation analysis [28,29,30], complex grayscale fluctuations are transformed into repeatable and comparable indicators, including activity intensity, apparent interfacial renewal, high-frequency fraction, burst-event rate, and apparent propagation characteristics [31,32]. This provides a physically interpretable analysis pathway for non-contact characterization of falling-film flow over tube bundles using large high-speed image datasets.

Based on the above considerations, this study uses high-speed reflective images of falling-film flow over a five-row horizontal tube bundle as the analysis target and establishes an image-based proxy-descriptor framework for state comparison. Under consistent optical conditions, the method transforms local grayscale fluctuations into descriptors such as the normalized spatiotemporal response, dynamic renewal field, activity intensity, frequency composition, and burst events. These descriptors are used to characterize differences in apparent interfacial activity among Reynolds numbers and tube-row positions, rather than to directly reconstruct film thickness, wave height, or interfacial velocity. The flow-state discrimination capability, robustness, and interpretive limits of the framework are then assessed through comparisons of typical flow states, descriptor distributions across tube rows, zero-response baseline tests, parameter-sensitivity analyses, noise correction, and event-threshold analyses. The proposed framework provides a non-contact visual monitoring approach with a relatively low calibration burden, repeatable processing, and compatibility with large high-speed image sequences, together with relative quantitative indicators for complex falling-film flows over tube bundles.

## 2. Experimental Setup and High-Speed Imaging System

### 2.1. Experimental Setup and Illumination Arrangement

The high-speed visualization data used in this study were obtained from an isothermal horizontal tube-bundle falling-film test rig. The fluid circuit and structural design of the test rig have been described in detail in previous work [33]; therefore, only the core configurations relevant to image sensing and subsequent processing are presented here.

Deionized water was used as the working fluid, and the fluid temperature was maintained at 20 ± 0.2 °C using a constant-temperature control unit. The working fluid was delivered by a circulation pump to a high-level tank and then passed through a turbine flowmeter with an accuracy of ±1.0% before entering the distributor at the top of the tube bundle. The distributor consisted of two closely arranged smooth copper tubes, with a single row of evenly spaced orifices machined along the bottom. The orifice diameter was 3 mm, the pitch was 10 mm, and the effective distribution length was 300 mm, providing a relatively uniform initial liquid supply at the inlet of the test section.

The tube-bundle test section consisted of five rows of horizontal copper tubes. Each tube had an effective length of 300 mm, an outer diameter of 21.3 mm, and a vertical inter-tube spacing of 12 mm. The film Reynolds number was defined as ${Re}_{\Gamma }=4\Gamma /\mu$, where Γ is the mass flow rate per unit length on one side of the tube and μ is the dynamic viscosity of the working fluid. The ${Re}_{\Gamma }$ range covered in the experiments was 184–960, covering the typical flow-regime transition from column flow to sheet flow.

**Figure 2:**
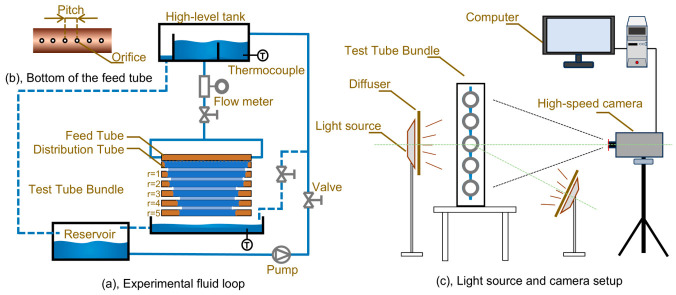
Experimental apparatus and high-speed imaging arrangement for falling-film flow over a horizontal tube bundle. (**a**) Closed-loop water circuit, including the reservoir, pump, high-level tank, flowmeter, distributor, and five-row horizontal tube bundle. (**b**) Bottom view of the feed tube showing the orifice arrangement used to distribute the liquid onto the tube bundle. (**c**) Optical arrangement of the high-speed imaging system, including the bilateral diffused LED illumination and the camera viewing direction.

As shown in Figure 2, the high-speed imaging system employed two 200 W LED light sources with diffusers for illumination. The light sources were placed on the front and rear sides of the tube bundle, respectively, to provide stable diffuse illumination. This bilateral illumination provided sufficient grayscale contrast for the liquid film, which served as the basis for subsequent fluctuation-feature extraction. The falling-film flow process was recorded using a high-speed camera (NAC Image Technology, Tokyo, Japan) with an image resolution of 1920 × 1080 pixels and an acquisition frame rate of 2000 fps. At this frame rate, the Nyquist frequency is 1000 Hz, covering the low- to medium-frequency interfacial fluctuation features mainly considered in this study. Spatial calibration showed that the physical scale corresponding to a single pixel was approximately 0.23 mm/px.

**Figure 3:**
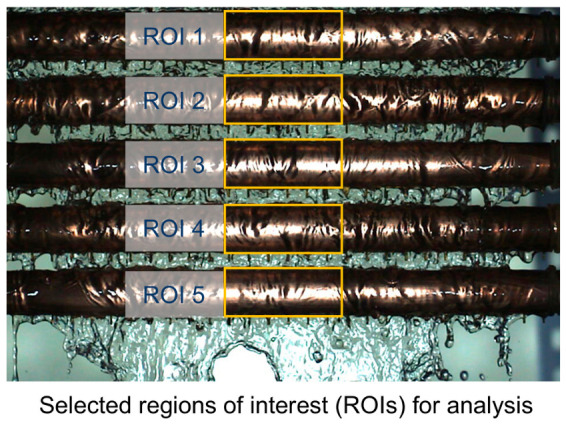
Fixed row-wise regions of interest for visual-proxy analysis. The yellow rectangles indicate the five ROIs selected at the same relative position on each tube row.

For each operating condition, 2000 consecutive raw grayscale images were acquired, corresponding to a recording duration of 1 s. This recording length was selected to capture the dominant interfacial renewal and fluctuation events within a consistent statistical window. As shown in Figure 3, a rectangular region of interest (ROI) with a fixed size was selected at the same relative position on each tube row. Each ROI had dimensions of 220 × 90 pixels, corresponding to approximately 50.6 mm × 20.7 mm. One ROI was selected from each of the five tube rows, yielding five analysis windows in total. For all operating conditions, the ROIs were defined using the same geometric reference positions of the tube bundle and maintained identical relative positions and dimensions, ensuring comparability across different flow-rate conditions. In this study, the high-speed imaging system was used as a non-contact optical sensing unit. The raw sensing signal was defined as the grayscale intensity field I(x,y,t) of each pixel within the ROI, and all subsequent spatiotemporal feature extraction used this raw grayscale field as the input.

### 2.2. Overview of the Image-Processing Workflow

To obtain comparable local dynamic features, the grayscale image sequences acquired by the high-speed imaging system were treated as non-contact visual sensing signals. For the ROI corresponding to the i-th tube row, the raw input was defined as the local grayscale field $I_{i}(x,y,t)$. With consistent imaging perspective, exposure parameters, illumination arrangement, and ROI definition, grayscale perturbations under different operating conditions can be used to characterize relative changes in the apparent interfacial activity of the liquid film.

**Figure 4:**
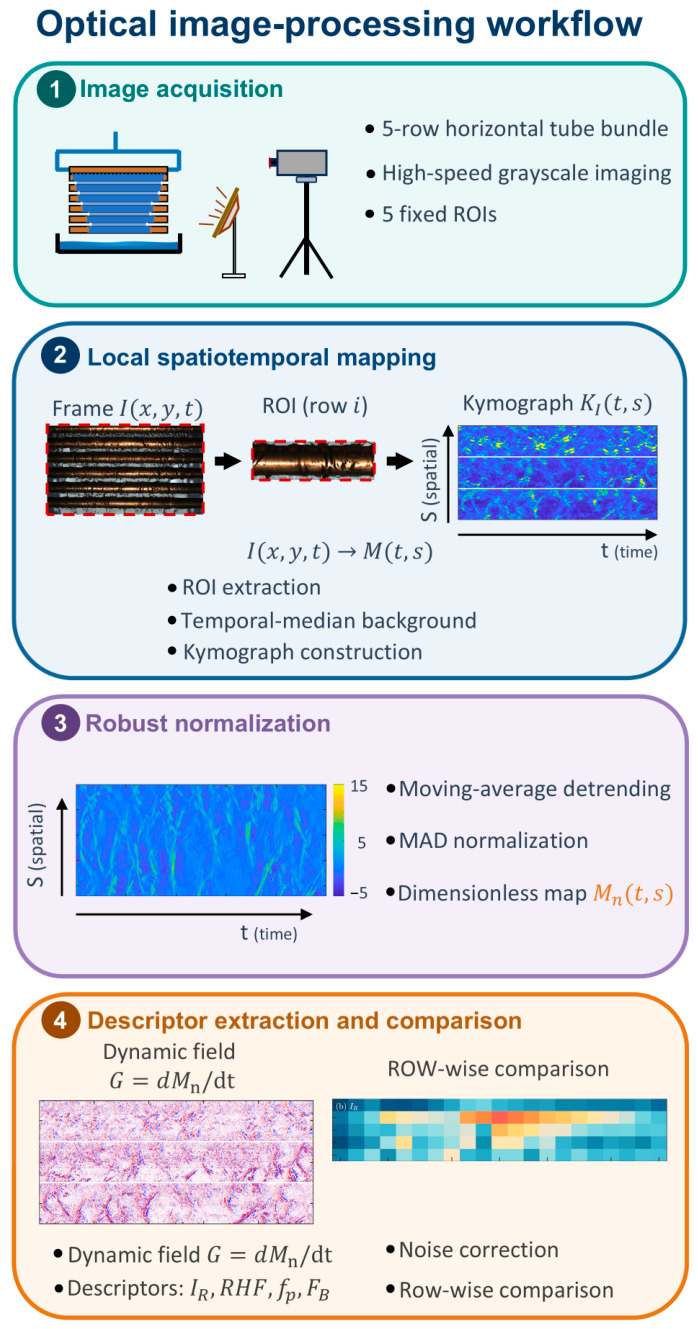
Optical image-processing workflow for visual-proxy sensing of falling-film flow over a horizontal tube bundle. Step 1: high-speed grayscale image acquisition under fixed optical and ROI settings. Step 2: ROI extraction, temporal-median background subtraction, and local spatiotemporal mapping. Step 3: moving-average detrending and MAD-based robust normalization to obtain the normalized spatiotemporal map $M_{n}$. Step 4: construction of the dynamic renewal field G and extraction of scalar optical-proxy descriptors, including $I_{R}$, $R_{HF}$, $f_{p}$, and $F_{B}$, for row-wise and condition-wise comparison. Colors in the schematic maps indicate the magnitude of the normalized or dynamic optical response; red and blue in G represent positive and negative temporal changes, respectively.

The image-processing workflow established in this study is illustrated in Figure 4. First, a temporal-median background is constructed and subtracted from the raw image sequence to obtain a local apparent activity field. The activity field is then line-averaged within the ROI to form a spatiotemporal map $M_{i}(t,s)$. On this basis, moving-average detrending and MAD-based robust normalization are applied to obtain a dimensionless spatiotemporal map $M_{n,i}(t,s)$, from which the dynamic renewal field $G_{i}(t,s)$ is further constructed. Finally, optical-proxy descriptors, including the apparent interfacial renewal intensity $I_{R}$, high-frequency fraction $R_{HF}$, dominant frequency $f_{p}$, and burst-event rate $F_{B}$, are extracted from the normalized spatiotemporal map and its dynamic renewal field. These descriptors are used to compare local activity differences among different Reynolds numbers and tube-row positions. The mathematical definitions, parameter settings, and physical interpretation of each processing step are presented in detail in Section 3. $M_{n}$, G, and all scalar descriptors are defined as image-derived proxies under fixed optical conditions and are intended primarily for relative comparison under consistent experimental conditions.

## 3. Optical-Proxy Descriptor Methodology for High-Speed Reflective Images

### 3.1. Local Spatiotemporal Mapping and Background Subtraction

In this study, a fixed-size local observation window is first defined at the corresponding position of each tube row, and a local spatiotemporal map is then constructed based on this window. Let the raw grayscale image sequence be

(1) $$I\left(x,y,t_{k}\right),k=1,2,\ldots ,N_{t}$$

where $t_{k}$ denotes the time instant corresponding to the k-th frame, and x and y represent the axial and vertical coordinates in the image plane, respectively. The tube-row index is denoted by i. Because the liquid-film activity among different tube rows is mainly reflected in the spatiotemporal evolution of local reflective structures, and the full image contains background regions that are not relevant to local-state characterization, the ROI is adopted as the basic analysis unit. To ensure sampling consistency across tube rows and operating conditions, the centers of all ROIs are fixed on the vertical centerline of the tube bundle, and the same ROI dimensions are maintained.

To mitigate the influence of static wall textures, fixed reflective patterns, and non-flow background on the characterization of image-based activity, a background frame set B is uniformly sampled from the raw image sequence to construct a temporal-median background image:

(2) $$I_{bg}\left(x,y\right)={median}_{t_{j}\in B}\left[I\left(x,y,t_{j}\right)\right]$$

Subsequently, within the i-th ROI, the absolute difference between the current frame and the background image is defined as the local apparent activity field:

(3) $$A_{i}\left(x,y,t_{k}\right)=|I\left(x,y,t_{k}\right)-I_{bg}\left(x,y\right)|,\left(x,y\right)\in \Omega_{i}$$

where $A_{i}(x,y,t_{k})$ characterizes the local grayscale activity magnitude relative to the static background. This activity is jointly affected by interfacial morphological changes, surface-slope variations, and local reflection responses. The absolute value is used to preserve the magnitude of deviation of local reflective structures from the background and to avoid cancellation between brightness enhancement and dimming in subsequent statistical analyses.

Based on the two-dimensional apparent activity field, the local image activity within the ROI is further reduced to a one-dimensional spatial profile and stacked over successive frames to form a spatiotemporal map. Let s be the discrete spatial coordinate along the primary analysis direction of the ROI, and let η be the coordinate perpendicular to that direction. The local spatiotemporal map is then defined as

(4) $$M_{i}\left(t_{k},s\right)={\left\langle A_{i}\left(s,\eta ,t_{k}\right)\right\rangle }_{\eta }$$

where ${\left\langle \cdot \right\rangle }_{\eta }$ denotes the line-averaging operation along the gravitational flow direction. Thus, s corresponds to the horizontal pixel position within the ROI. Equivalently, the above expression can be written as

(5) $$M_{i}\left(t_{k},s\right)=\frac{1}{N_{\eta }}\sum_{\eta =1}^{N_{\eta }}A_{i}\left(s,\eta ,t_{k}\right)$$

where $N_{\eta }$ is the number of pixels along the vertical averaging direction. The resulting$\;M_{i}\;(t_{k},s)$ represents the local reflective activity as a two-dimensional time–space map and is used to characterize spatiotemporal features such as local activity propagation, streak organization, localized activity enhancement, and intermittent intensification.

### 3.2. Robust Normalization and Dynamic Renewal Field Construction

To reduce the influence of local illumination distribution, static reflection-intensity differences, and slow brightness drift, temporal detrending and robust normalization are further applied after background subtraction. This step converts the spatiotemporal map $M_{i}\;(t_{k},s)$ into a dimensionless normalized spatiotemporal map $M_{n,i}\;(t_{k},s)$, thereby reducing the effects of slowly varying optical bias and anomalously bright reflections on the statistical scale.

At each spatial position s, a moving average is applied along the temporal direction and used as the local slowly varying baseline. This operation removes possible low-frequency drift components in $M_{i}\;(t_{k},s)$. Let the detrending window length be $T_{w}$, and the corresponding number of frames be

(6) $$N_{w}=round\left(\frac{T_{w}}{\Delta t}\right)$$

where Δt is the time interval between two consecutive frames. The slowly varying baseline for the i-th ROI is then expressed as

(7) $$B_{i}\left(t_{k},s\right)={movmean}_{N_{w}}\left[M_{i}\left(t_{k},s\right)\right]$$

and the detrended spatiotemporal map is defined as

(8) $$M_{0,i}\left(t_{k},s\right)=M_{i}\left(t_{k},s\right)-B_{i}\left(t_{k},s\right)$$

Here, $B_{i}\;(t_{k},s)$ denotes the slowly varying baseline obtained by temporal moving averaging, and $M_{0,i}\left(t_{k},s\right)$ represents the local activity perturbation after low-frequency drift removal. In this study, $T_{w}=0.10\;s$, which corresponds to approximately 200 frames at a sampling frequency of $f_{s}=2000\;Hz$.

To avoid the excessive influence of non-Gaussian noise, local specular highlights, and sporadic strong reflections on mean–standard-deviation normalization, a robust standardization method based on the median and the median absolute deviation (MAD) is adopted. For each spatial position s, the temporal median of the detrended time series $M_{0,i}\left(t_{k},s\right)$ is first computed as

(9) $$\mu_{i}\left(s\right)={median}_{k}\left[M_{0,i}\left(t_{k},s\right)\right]$$

together with the corresponding median absolute deviation,

(10) $${MAD}_{i}\left(s\right)={median}_{k}\left[|M_{0,i}\left(t_{k},s\right)-\mu_{i}\left(s\right)|\right]$$

The normalized spatiotemporal map is then defined as

(11) $$M_{n,i}\left(t_{k},s\right)=\frac{M_{0,i}\left(t_{k},s\right)-\mu_{i}\left(s\right)}{1.4826\;{MAD}_{i}\left(s\right)+\varepsilon }$$

where ε is a small positive number introduced to avoid division by zero; in this study, $\varepsilon =10^{-6}$. The coefficient 1.4826 makes the MAD-based scale consistent with the standard deviation under approximately Gaussian conditions. The resulting $M_{n,i}\left(t_{k},s\right)$ is dimensionless and indicates the deviation of the local apparent activity from the robust statistical scale at the corresponding spatial position.

Compared with the original spatiotemporal map $M_{i}\left(t_{k},s\right)$, the normalized map $M_{n,i}\left(t_{k},s\right)$ is less sensitive to local strong highlights, asymmetric disturbances, and a small number of abnormal frames. It is therefore more suitable for representing liquid-film activity signals with evident non-Gaussian characteristics in high-speed reflective images. Meanwhile, the normalization reduces spatial bias caused by non-uniform illumination and fixed reflection-intensity differences within the ROI, while retaining the relative temporal variations in the activity structures.

### 3.3. Baseline Descriptor Definition

To construct repeatable and comparable image-domain proxies for each tube row, baseline descriptors of local apparent liquid-film activity are extracted from three aspects: dynamic intensity, frequency composition, and intermittency. These descriptors are used to characterize relative differences in local reflective activity under different operating conditions, tube-row positions, and structural configurations.

To quantify the rapid temporal renewal of the local apparent activity pattern, a temporal low-pass filter is first applied to the normalized spatiotemporal map $M_{n,i}\left(t_{k},s\right)$. This step reduces the amplification of high-frequency noise during numerical differentiation. Let the low-pass filtered normalized spatiotemporal map be denoted by ${\tilde{M}}_{n,i}\left(t_{k},s\right)$. The dynamic renewal field is then defined by forward differencing as

(12) $$G_{i}\left(t_{k+\frac{1}{2}},s\right)=\frac{{\tilde{M}}_{n,i}\left(t_{k+1},s\right)-{\tilde{M}}_{n,i}\left(t_{k},s\right)}{\Delta t},k=1,2,\ldots ,N_{t}-1$$

Here, $\Delta t=1/f_{s}$ is the time interval between two consecutive frames, and $f_{s}$ is the sampling frequency. Because $G_{i}$ is obtained from the forward difference between adjacent frames, its temporal location is

(13) $$t_{k+\frac{1}{2}}=t_{k}+\frac{\Delta t}{2}$$

Therefore, $G_{i}$ represents the temporal rate of change of the normalized apparent activity pattern, or the renewal intensity of local reflective structures, rather than the true propagation velocity of the liquid-film interface. In this study, the low-pass cutoff frequency before differencing is denoted by $f_{c}LP$, which controls the effective temporal scales retained before the derivative operation.

The uncorrected apparent renewal intensity is then calculated as

(14) $$I_{R,meas,i}={\left[\frac{1}{(N_{t}-1)N_{s}}\sum_{k=1}^{N_{t}-1}\sum_{j=1}^{N_{s}}G_{i}^{2}\left(t_{k+\frac{1}{2}},s_{j}\right)\right]}^{1/2}$$

where $N_{s}$ is the number of spatial sampling points along the main analysis direction of the ROI. This quantity is the root-mean-square value of the dynamic renewal field $G_{i}$, and is used to describe the overall dynamic level of local apparent activity within a given ROI.

However, temporal differencing amplifies high-frequency noise. To reduce the influence of camera noise and high-frequency optical disturbances on $I_{R,meas,i}$, a frequency-domain noise-floor correction based on the Parseval relation is further introduced. For each spatial position $s_{j}$, the one-sided power spectral density of $M_{n,i}\left(t,s_{j}\right)$, denoted by $S_{M_{n},i,j}(f)$, is calculated. The contribution of the noise component after the processing chain of noise-band selection, low-pass filtering, and temporal differencing is then estimated.

Because the dynamic renewal field $G_{i}$ is obtained from the first-order temporal difference of $M_{n,i}$, the frequency response of the temporal-difference operator must be included when estimating the noise contribution to $I_{R}$. For the forward-difference scheme used here, the squared magnitude response is

(15) $${\left|H_{diff}(f)\right|}^{2}={\left[\frac{2}{\Delta t}sin\left(\pi f\Delta t\right)\right]}^{2}={\left[2f_{s}sin\left(\frac{\pi f}{f_{s}}\right)\right]}^{2}$$

Here, $H_{diff}(f)$ is the frequency response of the first-order temporal-difference operator, and $\Delta t=1/f_{s}$. At low frequencies, $H_{diff}(f)\approx 2\pi f$, which is consistent with the frequency response of the continuous-time derivative. At higher frequencies, this response increases, indicating that temporal differencing amplifies high-frequency noise. Including this term in the noise-floor correction accounts for the noise amplification introduced by numerical differentiation.

Let $H_{LP}(f)$ denote the frequency response of the low-pass filter applied before differencing, and $H_{HP}(f)$ denote the high-pass weighting function used to select the high-frequency noise band. The contribution of noise to the square of the apparent renewal intensity is defined as

(16) $$I_{R,noise,i}^{2}=\frac{1}{N_{s}}\sum_{j=1}^{N_{s}}\int_{0}^{f_{N}}S_{M_{n},i,j}\left(f\right){|H_{HP}\left(f\right)|}^{2}{|H_{LP}\left(f\right)|}^{2}{|H_{diff}\left(f\right)|}^{2}\;df$$

where $f_{N}=f_{s}/2$ is the Nyquist frequency. In this study, $H_{HP}(f)$ is gradually activated using a cosine transition around the noise-band starting frequency $f_{n,start}$, and is used to estimate the high-frequency noise component. $H_{LP}(f)$ is consistent with the low-pass filter used before constructing $G_{i}$. The noise-corrected apparent renewal intensity is finally defined as

(17) $$I_{R,i}=\sqrt{max\left(I_{R,meas,i}^{2}-I_{R,noise,i}^{2},0\right)}$$

The quantity $I_{R}$, with units of $s^{-1}$, represents the noise-corrected temporal renewal intensity of the normalized image-activity field. A larger value indicates stronger temporal variation in the local reflective activity pattern.

Second, to describe the proportion of rapidly varying components in the local activity signal, frequency-composition descriptors are defined directly from the normalized activity pattern. Unlike $I_{R}$, these frequency-related descriptors are not calculated from the dynamic renewal field $G_{i}$, but from the spatially averaged signal of the normalized spatiotemporal map:

(18) $${\bar{M}}_{n,i}\left(t_{k}\right)=\frac{1}{N_{s}}\sum_{j=1}^{N_{s}}M_{n,i}\left(t_{k},s_{j}\right)$$

The one-sided power spectral density of ${\bar{M}}_{n,i}\left(t_{k}\right)$, denoted by $S_{{\bar{M}}_{n,i}}(f)$, is then calculated. Let $f_{c}$ be the cutoff frequency used to distinguish rapidly varying components. The high-frequency fraction is defined as

(19) $$R_{HF,i}=\frac{\int_{f_{c}}^{f_{N}}S_{{\bar{M}}_{n},i}(f)\;df}{\int_{0}^{f_{N}}S_{{\bar{M}}_{n},i}(f)\;df}$$

The dominant frequency is defined as the frequency corresponding to the peak of the power spectral density:

(20) $$f_{p,i}=\underset{f}{arg\;max}S_{{\bar{M}}_{n},i}\left(f\right)$$

Here, $R_{HF}$ represents the fraction of rapidly varying components above $f_{c}$ in the normalized apparent activity signal, whereas $f_{p}$ characterizes the dominant temporal scale of this signal. Spectral analysis is performed on ${\bar{M}}_{n,i}(t)$, rather than on ${\bar{G}}_{i}(t)$, to avoid the intrinsic high-frequency weighting introduced by temporal differencing. In this way, $R_{HF}$ and $f_{p}$ more directly reflect the frequency composition of the original normalized activity pattern. Although the processing script also retains frequency metrics based on $G_{i}$ as reference outputs, the high-frequency fraction and dominant frequency discussed in this paper refer to the results based on ${\bar{M}}_{n,i}$.

Third, to characterize the occurrence frequency of strong burst-like renewal events, the spatially averaged signal of the dynamic renewal field is constructed as

(21) $${\bar{G}}_{i}\left(t_{k+\frac{1}{2}}\right)=\frac{1}{N_{s}}\sum_{j=1}^{N_{s}}G_{i}\left(t_{k+\frac{1}{2}},s\right)$$

The event-detection threshold is defined based on the standard deviation of this signal:

(22) $$\Theta_{E,i}=\kappa \sigma_{\bar{G},i},$$

where $\sigma_{\bar{G},i}$ is the standard deviation of ${\bar{G}}_{i}$, and κ is the threshold coefficient. In this study, κ=3. A binary event sequence is then defined as

(23) $$Q_{i}\left(k\right)=\left\{\begin{array}{cc} 1, & |{\bar{\;G}}_{i}\left(t_{k+\frac{1}{2}}\right)|>\Theta_{E,i},\\ 0, & |{\bar{\;G}}_{i}\left(t_{k+\frac{1}{2}}\right)|\leq \Theta_{E,i}. \end{array}\right.$$

To avoid repeated counting of the same continuous supra-threshold interval, only the rising edge from 0 to 1 in the binary event sequence is counted as one independent strong apparent renewal event. The burst-event rate is therefore defined as

(24) $$F_{B,i}=\frac{1}{T_{G}}\sum_{k=1}^{N_{t}-1}I\left[Q_{i}(k)=1,\;Q_{i}(k-1)=0\right]$$

where I[⋅] is the indicator function, $Q_{i}(0)=0$, and $T_{G}=(N_{t}-1)\Delta t$ is the duration of the dynamic renewal signal. $F_{B,i}$ characterizes whether strong-renewal processes occur in a discrete, burst-like, and intermittent manner. In some flow states, even when the overall dynamic intensity is not the highest, $F_{B,i}$ may remain high if the local activity is dominated by a small number of strong burst-like reorganization events.

In summary, an image-based proxy characterization system is constructed from the normalized spatiotemporal map $M_{n,i}(t,s)$. The field quantities include the normalized spatiotemporal map $M_{n,i}(t,s)$ and the dynamic renewal field $G_{i}(t,s)$, which describe the spatiotemporal organization, streak structures, intermittent enhancement, and spatial concentration of local reflective activity. The scalar descriptors include the noise-corrected apparent renewal intensity $I_{R,i}$, high-frequency fraction $R_{HF,i}$, dominant frequency $f_{p,i}$, and burst-event rate $F_{B,i}$, corresponding to dynamic intensity, frequency composition, dominant temporal scale, and intermittency, respectively. For the ROI corresponding to the i-th tube row, the baseline descriptor vector is written as

(25) $$D_{i}=\left[I_{R,i},\;R_{HF,i},\;f_{p,i},\;F_{B,i}\right]$$

Here, $I_{R,i}$ represents the overall temporal renewal intensity of the local reflective activity pattern, $R_{HF,i}$ represents the relative contribution of rapid perturbation components in the normalized activity signal, $f_{p,i}$ represents the dominant temporal scale of the identifiable response, and $F_{B,i}$ represents the occurrence frequency of strong burst-like apparent renewal events. These descriptors provide a unified quantitative basis for comparing apparent liquid-film activity under different Reynolds numbers and tube-row positions. The definitions and interpretations of these field quantities and scalar descriptors are summarized in Table 2.

### 3.4. Uncertainty and $Re_{\Gamma }$-Propagation Analysis

To evaluate the influence of flow-rate measurement uncertainty on the image-derived descriptors, the uncertainty of $Re_{\Gamma }$ was propagated to the descriptor uncertainty. The film Reynolds number is defined as

(26) $$Re_{\Gamma }=\frac{4\Gamma }{\mu_{l}}$$

where Γ is the one-sided liquid-film mass flow rate per unit width, and $\mu_{l}$ is the dynamic viscosity of the liquid phase. When the measured quantity is the total mass flow rate supplied to the tube bundle, Γ is calculated by assuming two-sided film formation. The standard uncertainty of $Re_{\Gamma }$ is determined from the uncertainties in flow-rate measurement and liquid viscosity:

(27) $${\left(\frac{u(Re_{\Gamma })}{Re_{\Gamma }}\right)}^{2}={\left(\frac{u(\Gamma )}{\Gamma }\right)}^{2}+{\left(\frac{u(\mu_{l})}{\mu_{l}}\right)}^{2}$$

The expanded uncertainty is calculated using a coverage factor of k=2:

(28) $$U_{95}\left(Re_{\Gamma }\right)=2u\left(Re_{\Gamma }\right)$$

Under the present experimental conditions, the relative expanded uncertainty of $Re_{\Gamma }$ is approximately 0.64%, which is smaller than the intrinsic scatter of the image-derived descriptors. For each operating condition, the descriptors from the five ROIs are averaged to obtain a bundle-scale descriptor:

(29) $$\bar{D}=\frac{1}{N}\sum_{i=1}^{N}\;D_{i},N=5$$

where D represents $I_{R}$, $R_{HF}$, $f_{p}$, or $F_{B}$. The uncertainty of the bundle-scale descriptor includes image-processing uncertainty, spatial variation among ROIs, and the uncertainty propagated from $Re_{\Gamma }$:

(30) $$u^{2}\left(\bar{D}\right)=u_{img}^{2}\left(\bar{D}\right)+u_{ROI}^{2}\left(\bar{D}\right)+u_{Re}^{2}\left(\bar{D}\right)$$

Here, $u_{img}(\bar{D})$ denotes the uncertainty introduced by the image-processing procedure, and $u_{ROI}(\bar{D})$ denotes the spatial scatter among different ROIs. The $Re_{\Gamma }$-propagated term is evaluated from the local sensitivity:

(31) $$u_{Re}\left(\bar{D}\right)=\left|\frac{\partial \bar{D}}{\partial Re_{\Gamma }}\right|u\left(Re_{\Gamma }\right)$$

To quantify the contribution of $Re_{\Gamma }$ uncertainty to the total descriptor uncertainty, the following ratio is defined:

(32) $$C_{Re}=100\frac{u_{Re}^{2}(\bar{D})}{u^{2}(\bar{D})}$$

Table 3 presents the uncertainty-propagation results for the bundle-scale descriptors. The relative expanded uncertainty is defined as

(33) $$\frac{100U_{95}(\bar{D})}{\left|\bar{D}\right|}$$

The results show that the median relative expanded uncertainties of the four image-derived descriptors are suitable for comparative trend analysis. Among them, $I_{R}$ has the lowest uncertainty, with a median relative expanded uncertainty of 11.35%. The corresponding values for $R_{HF}$, $f_{p}$, and $F_{B}$ are 24.05%, 29.63%, and 25.95%, respectively. These values indicate that the descriptors are affected by local flow non-uniformity and image-processing uncertainty, while their overall uncertainty level remains acceptable for comparing trends under different $Re_{\Gamma }$ conditions.

The contribution of $Re_{\Gamma }$ uncertainty to the total descriptor variance is small. For $I_{R}$, $R_{HF}$, $f_{p}$, and $F_{B}$, the maximum values of $C_{Re}$ are 2.00%, 0.33%, 2.08%, and 0.17%, respectively. In the present experiments, the descriptor uncertainty is mainly associated with ROI-to-ROI spatial variation or image-processing uncertainty. The contribution propagated from flow-rate measurement uncertainty through $Re_{\Gamma }$ is limited.

This uncertainty budget supports the use of $I_{R}$, $R_{HF}$, $f_{p}$, and $F_{B}$ for discussing changes in liquid-film morphology and dynamic behavior with $Re_{\Gamma }$. The measurement uncertainty of $Re_{\Gamma }$ has been included in the error budget, and its contribution is much smaller than the spatial and processing uncertainties of the image-derived descriptors. It has only a minor influence on the present assessment of flow-regime evolution and interfacial dynamic features. The proposed workflow is deterministic for a fixed image sequence, ROI definition, and processing-parameter set, and thus the descriptor values are repeatable at the processing level. However, run-to-run repeatability can still be influenced by inlet distribution, initial wetting state, surface condition, ROI registration, camera alignment, and illumination stability. Background removal, robust normalization, and noise correction reduce the effect of static illumination non-uniformity and stationary noise, but temporal light drift, flicker, specular saturation, defocusing, and viewing-angle changes may still affect descriptor magnitudes. Therefore, all comparisons in this study are made under fixed optical settings, and the descriptors are used as comparative optical proxies rather than absolute physical measurements.

## 4. Typical Flow States: Visual Characterization and Descriptor Response

### 4.1. Representative Visual States and Their Spatiotemporal Signatures

**Figure 5:**
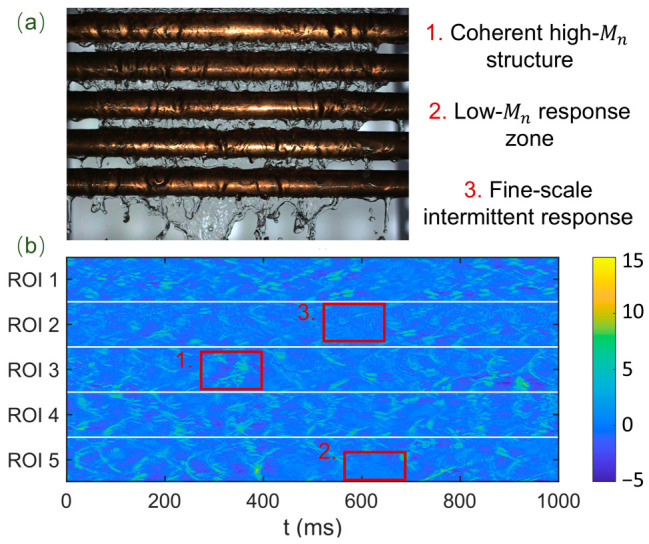
Row-wise differences in local visual responses under the same falling-film condition. (**a**) Raw high-speed image of the five-row tube bundle. (**b**) Corresponding normalized spatiotemporal map $M_{n}$ constructed from ROI1–ROI5, 1. coherent high-$M_{n}$ structure, 2. low-$M_{n}$ response zone, and 3. fine-scale intermittent response. The color scale denotes the dimensionless normalized optical fluctuation intensity.

To evaluate the ability of the image-processing framework to characterize local flow states, this section first compares the visualization images and the corresponding processed results at different tube-row positions, and then examines the variation in local spatiotemporal textures for the entire tube bundle and for a representative tube row at different Reynolds numbers. Figure 5 shows the visualization image of the five-row tube bundle under the same operating condition, together with the corresponding processed result. The raw image shows distinct local flow morphologies among the tube rows, while the processed normalized spatiotemporal map $M_{n}$ converts these differences into identifiable spatiotemporal texture features.

The liquid film does not remain spatially uniform during its downstream evolution, but exhibits different fluctuation patterns at different tube-row positions. Three representative local features can be identified in Figure 5. The first is a continuous high-response structure, appearing as an extended region with elevated $M_{n}$ values and corresponding to a locally concentrated activity pattern. The second is a low-response region, where local fluctuations remain weak over a relatively long period, indicating limited grayscale variation at that position. The third is a fine-scale intermittent response structure, appearing as dispersed and fragmented high-value textures, suggesting more transient and discrete local activity. The processed spatiotemporal map preserves the main flow differences among tube rows and highlights local feature patterns that are difficult to quantify directly from the raw image. It therefore provides a basis for comparing local activity intensity, continuity, and intermittency among different tube rows.

**Figure 6:**
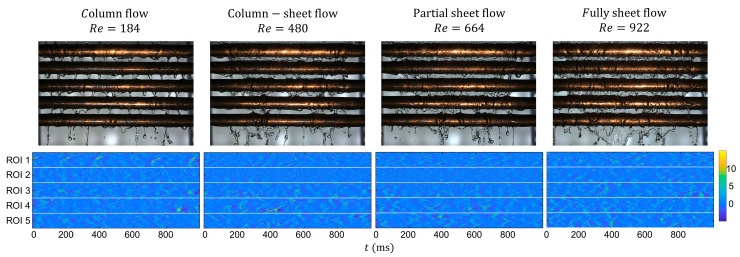
Correspondence between expert-reference falling-film states and normalized spatiotemporal responses at different Reynolds numbers. The four columns correspond to CF, CSF, PF, and FSF, respectively. The upper panels show representative raw visualization images, and the lower panels show the corresponding row-wise normalized spatiotemporal maps $M_{n}$ for ROI1–ROI5.

After clarifying the local response differences among tube-row positions, Figure 6 further compares the high-speed images and the corresponding $M_{n}$ maps at different $Re_{\Gamma }$ values at the tube-bundle scale. As $Re_{\Gamma }$ increases, the falling-film flow over the tube bundle gradually changes from a relatively discrete column-flow wetting state at low $Re_{\Gamma }$ to a sheet-flow state with larger coverage and more evident inter-row liquid connectivity. Correspondingly, the $M_{n}$ maps change from weak and sparsely distributed low-activity textures to denser and more continuous high-response structures, accompanied by stronger local streaks and expanded active regions. This comparison indicates that, although $M_{n}$ does not directly represent the actual film thickness or wave height, it can capture relative changes in the overall wetting morphology, local fluctuation enhancement, and inter-row non-uniformity under consistent optical conditions. In this way, the flow-state evolution observed in the raw high-speed images is converted into comparable spatiotemporal responses.

After clarifying the local response differences among tube-row positions, Figure 6 compares the high-speed images and the corresponding $M_{n}$ maps at different $Re_{\Gamma }$ values at the tube-bundle scale. The four representative conditions correspond to the expert-reference states from column flow to fully developed sheet flow, namely CF, CSF, PF, and FSF. As $Re_{\Gamma }$ increases, the raw images show a gradual change from discrete column-dominated wetting to broader sheet-like coverage and stronger inter-row liquid connectivity. Correspondingly, the $M_{n}$ maps evolve from sparse low-activity textures to denser and more continuous high-response structures with expanded active regions. This comparison shows that $M_{n}$, under consistent optical conditions, converts the visible flow-state evolution into comparable spatiotemporal responses.

Using Figure 6 as the visual reference, an expert-based flow-regime classification was introduced to relate the observed flow states to the processed spatiotemporal maps. Following previous studies on flow-regime transitions in falling films over horizontal tube bundles [33], the visible flow states were classified into four categories: column flow (CF), column-sheet flow (CSF), partial sheet flow (PF), and fully developed sheet flow (FSF). CF refers to a state dominated by discrete liquid columns, with no evident sheet-like regions. CSF describes the early merging of neighboring liquid columns, accompanied by local sheet-like regions or initially connected liquid regions. PF corresponds to a state in which sheet-like flow becomes more evident, while discontinuous coverage and residual columnar streams are still present. FSF represents a state in which the sheet-like liquid film covers most of the effective distribution width. An apparent sheet-coverage ratio above 90% was used as an auxiliary criterion for FSF.

During expert labeling, the flow state was first judged from the complete high-speed video sequence. The corresponding ROI-based $M_{n}$ spatiotemporal map was then examined to confirm whether the main dynamic structures showed a consistent response. The raw image in Figure 6 provides a representative morphological snapshot, and the $M_{n}$ map below it shows the continuous evolution within the recording window. When visually identifiable events, such as liquid-column merging, sheet spreading, local contraction, or intermittent strong fluctuation, appeared in $M_{n}$ as continuous streaks, localized high-response regions, or enhanced textures, the sample was regarded as visually consistent. This procedure was used to validate the spatiotemporal maps against direct expert annotations from the raw videos, without using the downstream scalar descriptors as the primary basis for labeling.

To provide quantitative support for the reference classification, three auxiliary image-derived metrics were extracted from $M_{n}$. The mean normalized optical fluctuation intensity $A_{M}$ describes the overall optical fluctuation level within the ROI. The strong-fluctuation spatiotemporal fraction $P_{M}$ characterizes local strong fluctuations and intermittent enhancement. The apparent sheet-coverage ratio $C_{S}$ describes the extent of sheet-like film spreading within the effective distribution width. These auxiliary metrics are defined as

(34) $$A_{M}=\frac{1}{TN_{s}}\sum_{t=1}^{T}\;\sum_{s=1}^{N_{s}}\;\left|M_{n}\left(t,s\right)\right|$$

(35) $$P_{M}=\frac{1}{TN_{s}}\sum_{t=1}^{T}\;\sum_{s=1}^{N_{s}}\;1\left(\left|M_{n}(t,s)|>\theta_{M}\right.\right)$$

(36) $$C_{s}=\frac{W_{s}}{W_{eff}}$$

where T is the number of temporal samples, $N_{s}$ is the number of spatial samples, $\theta_{M}$ is the unified threshold for strong fluctuations, $W_{s}$ is the apparent sheet-coverage width, and $W_{eff}$ is the effective distribution width. When $C_{s}\geq 0.90$, the flow state was taken as consistent with FSF. Because the transition from CF to FSF is gradual, the critical $Re_{\Gamma }$ between adjacent reference states was expressed as a transition interval between two neighboring experimental conditions, instead of a sharp universal boundary.

Table 4 summarizes the expert-based reference criteria and the auxiliary image-derived metrics. CF, CSF, PF, and FSF provide the reference flow-state labels, while $A_{M}$, $P_{M}$, and $C_{S}$ provide quantitative support for classification. This treatment links the visual expert annotations, the $M_{n}$ spatiotemporal maps, and the subsequent scalar descriptor analyses based on $I_{R}$, $R_{HF}$, $f_{p}$, and $F_{B}$, supporting comparative flow-state assessment under fixed optical conditions.

**Figure 7:**
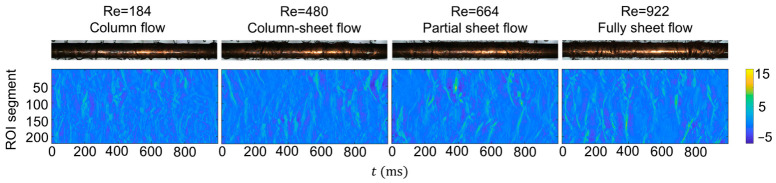
Evolution of local spatiotemporal textures in a representative tube row with increasing Reynolds number. The four columns correspond to ${Re}_{\Gamma }=184,\;480,\;664,\;$and 922. The upper strip shows the raw local image of the selected tube row, and the lower panel shows the corresponding normalized spatiotemporal map $M_{n}$.

Figure 7 further presents the visualization images and the corresponding processed results for the same representative tube row at different $Re_{\Gamma }$ values, based on the tube-bundle-scale comparison, to illustrate the response of the method to flow-state changes. As $Re_{\Gamma }$ increases from 184 to 922, the raw local images show a typical evolution from column flow to sheet flow, and the processed normalized spatiotemporal maps $M_{n}$ exhibit a corresponding texture evolution. Under the low- $Re_{\Gamma }$ column-flow condition, the processed maps are dominated by a low-amplitude background and sparse streak structures, indicating weak local activity and dispersed spatiotemporal variations. As the flow rate increases and enters the column-to-sheet transition regime, both the number of streaks and the extent of local high-value regions in the processed maps increase, suggesting enhanced local activity and a more evident spatial concentration of the response. With a further increase in $Re_{\Gamma }$, under partial and fully developed sheet-flow conditions, oblique streaks and local high-response structures become more distinct, and high-value textures appear more frequently. This indicates that the local activity gradually changes from sparse and intermittent perturbations to a more continuous and denser spatiotemporal organization.

Although the raw images of different flow states all show complex light–dark variations on the reflective liquid-film interface, unified processing converts the local-state differences into a common representation, making the spatiotemporal signatures directly comparable. The low-$Re_{\Gamma }$ state is characterized by weak activity, limited coverage, and sparse responses. The transition regime shows stronger local enhancement and more evident texture reorganization, whereas the sheet-flow state at higher $Re_{\Gamma }$ corresponds to a more continuous high-activity background and denser spatiotemporal structures. These observations are consistent with the expected physical trend. The processed maps can capture the overall evolution of local flow from discrete to continuous and from weak to stronger activity, providing a basis for the subsequent analysis of inter-row differences and descriptor-based state discrimination.

### 4.2. Response of Descriptors to Flow-State Evolution

**Figure 8:**
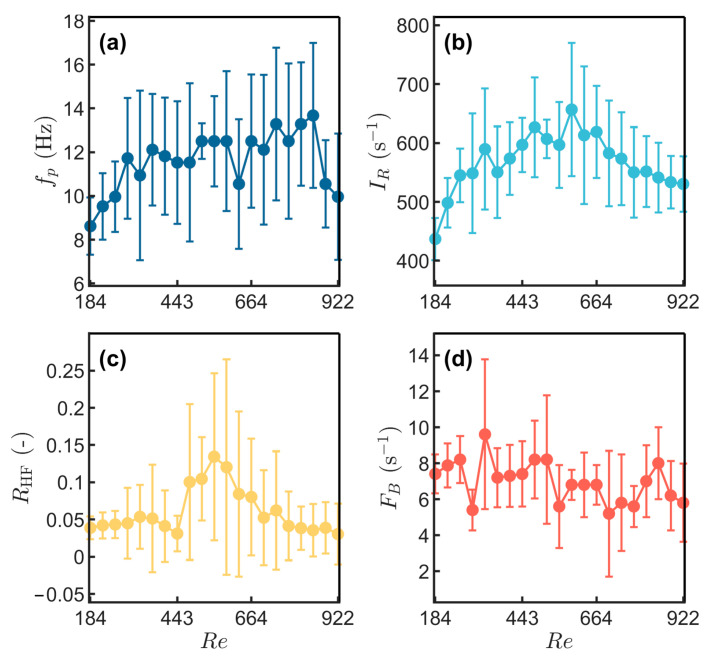
Descriptor responses to falling-film state evolution with increasing Reynolds number. (**a**) Dominant frequency $f_{p}$. (**b**) Noise-corrected apparent renewal intensity $I_{R}$. (**c**) High-frequency fraction $R_{HF}$. (**d**) Burst-event rate $F_{B}$. Symbols represent the mean value over the five ROIs, and error bars denote the standard deviation among ROIs. The error bars therefore indicate row-wise spatial dispersion of the image-derived descriptors rather than measurement uncertainty or repeatability error.

After establishing the correspondence between the raw images and the processed spatiotemporal signatures in the preceding section, this section further examines whether the baseline descriptors can convert different falling-film flow states into comparable quantitative responses. For each operating condition, the dominant frequency $f_{p}$, noise-corrected apparent renewal intensity $I_{R}$, high-frequency fraction $R_{HF}$, and burst-event rate $F_{B}$ are calculated for the five ROIs. The mean value across the five ROIs is then used to characterize the overall tube-bundle response under that condition. The error bars in Figure 8 represent the standard deviation among the five ROIs, which reflects the spatial dispersion across different tube-row positions. Therefore, these error bars indicate the non-uniformity of streamwise liquid redistribution and local apparent interfacial activity, rather than measurement uncertainty or repeatability error.

For the column-flow state at low $Re_{\Gamma }$, the liquid is transferred between tubes mainly in the form of discrete liquid columns or local liquid bridges. The local wetting coverage is limited, and the apparent interfacial activity is sparse and intermittent. Correspondingly, both $I_{R}$ and $R_{HF}$ remain at relatively low levels, indicating weak rapid renewal of local reflective structures and limited fine-scale high-frequency perturbations. Thus, the typical descriptor characteristics of the low- $Re_{\Gamma }$ state can be summarized as weak overall renewal intensity, limited high-frequency content, and intermittently occurring local events.

When $Re_{\Gamma }$ enters the intermediate range corresponding to the column-to-sheet transition regime, the descriptor responses show clear differentiation. At this stage, the liquid coverage increases, but a stable and continuous sheet structure has not yet fully formed. Local merging, impingement, breakup, rewetting, and reorganization of reflective structures become more frequent. Correspondingly, $I_{R}$ reaches a relatively high level in the intermediate $Re_{\Gamma }$ range, indicating stronger temporal renewal of the local apparent activity pattern. $R_{HF}$ also increases markedly, suggesting that rapid fine-scale perturbations account for a larger proportion of the overall activity. This combined response indicates that the intermediate transition regime is not merely a state of increased flow rate, but a regime with stronger local apparent interfacial activity and more complex spatiotemporal organization.

The error bars in Figure 8 further reveal inter-row differentiation at certain intermediate $Re_{\Gamma }$ values. Some tube rows exhibit stronger local renewal and higher high-frequency content, whereas others remain in a weaker or more intermittent activity state. The intermediate $Re_{\Gamma }$ range is therefore not only associated with a more pronounced overall dynamic response, but also with clearer spatial differences caused by streamwise liquid redistribution.

For the partial sheet-flow and fully developed sheet-flow states at higher $Re_{\Gamma }$, the liquid-film coverage is further increased, and the local apparent interfacial activity gradually shifts from discrete burst-like renewal toward a more continuous sheet-flow organization. At this stage, $I_{R}$ no longer increases monotonically with $Re_{\Gamma }$, but instead reaches a plateau or decreases slightly. $R_{HF}$ also decreases from the high values observed in the intermediate range. This indicates that the local variation pattern in the images has changed: the apparent activity is no longer dominated by strong discrete local reorganization events, but evolves more smoothly on a continuous coverage background.

The burst-event rate $F_{B}$ provides complementary information on this state transition. At low to moderate $Re_{\Gamma }$, local activity is more likely to appear as pronounced supra-threshold events, so $F_{B}$ can remain at a relatively high level. As the flow develops toward a continuous sheet-flow state, local renewal is gradually distributed into a more continuous background activity, and the relative contribution of strong burst-like events decreases. Consequently, $F_{B}$ decreases or tends to level off. Thus, under a unified threshold criterion, $F_{B}$ serves as an indicator of whether local apparent renewal has burst-like intermittent characteristics. A higher $F_{B}$ indicates that the renewal process relies more on discrete strong events, whereas a lower $F_{B}$ suggests a more continuous renewal process or event amplitudes insufficient to produce clear supra-threshold responses.

The dominant frequency $f_{p}$ plays a more auxiliary role. It represents the time scale associated with the main spectral peak in the normalized activity signal, rather than the true wave frequency in a strict sense. At low $Re_{\Gamma }$, lower $f_{p}$ values indicate slower local activity variations. In the intermediate $Re_{\Gamma }$ range, $f_{p}$ generally shifts toward higher frequencies, indicating a shorter local response time scale. At higher $Re_{\Gamma }$, the decrease or fluctuation of $f_{p}$ suggests that a higher single dominant frequency does not necessarily occur in the fully developed sheet-flow state. In this regime, local activity may appear as a more broadband and continuous background evolution, rather than being dominated by a clearly identifiable single frequency. Therefore, $f_{p}$ is more suitable as an auxiliary descriptor of the dominant time scale and should not be used alone for flow-state identification.

Taken together, these results show that the descriptors can convert the evolution of falling-film flow states—from discrete column flow, through transition-stage reorganization, to continuous sheet flow—into interpretable image-derived proxy responses.

### 4.3. Inter-Row State Differences and Descriptor Complementarity

**Figure 9:**
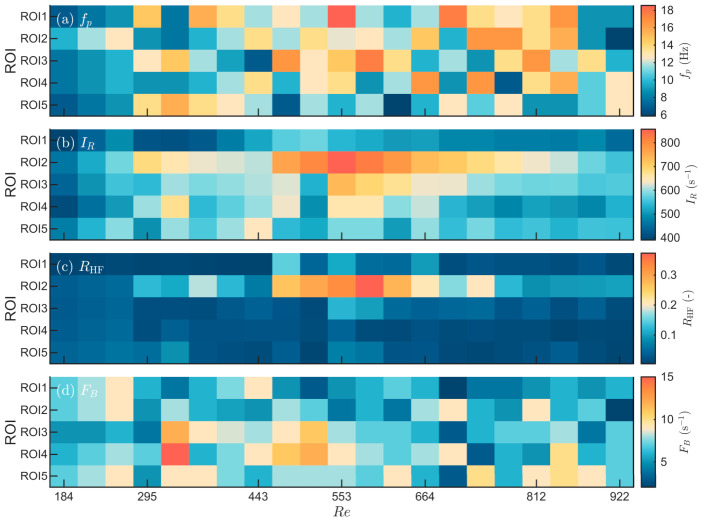
Row-wise distributions of visual-proxy descriptors at different Reynolds numbers. (**a**) Dominant frequency $f_{p}$. (**b**) Noise-corrected apparent renewal intensity $I_{R}$. (**c**) High-frequency fraction $R_{HF}$. (**d**) Burst-event rate $F_{B}$. The horizontal direction represents $Re_{\Gamma }$, and the vertical direction represents the ROI index from ROI1 to ROI5. The color scale indicates the magnitude of each image-derived descriptor.

To further examine the spatial distribution behind the overall statistical results, Figure 9 presents the two-dimensional distributions of $f_{p}$, $I_{R}$, $R_{HF}$, and $F_{B}$ as functions of $Re_{\Gamma }$ and ROI index. The descriptor maps show that the method can characterize not only the overall variation with $Re_{\Gamma }$, but also local-state differences among tube-row positions. In addition, the inter-row differences reflected by different descriptors are not identical. They correspond to different aspects of the local response, including the dominant time scale, renewal intensity, frequency composition, and intermittency.

First, Figure 9a shows the distribution of the dominant frequency $f_{p}$. The values of $f_{p}$ vary noticeably with both $Re_{\Gamma }$ and ROI position, but they do not exhibit a stable monotonic gradient along the tube rows. For example, at low $Re_{\Gamma }$, $f_{p}$ in the downstream rows can be higher than that in the upstream rows, whereas in the intermediate $Re_{\Gamma }$ range, local high values may appear at ROI1 or ROI4. This indicates that $f_{p}$ can reflect changes in the dominant time scale among tube rows, but its spatial distribution is relatively scattered. Therefore, $f_{p}$ is more suitable as an auxiliary descriptor and should not be used alone as the main basis for inter-row state discrimination.

In Figure 9b, $I_{R}$ exhibits a clearer spatial distribution pattern. At low $Re_{\Gamma }$, $I_{R}$ remains low across all rows, with limited inter-row differences. In the intermediate $Re_{\Gamma }$ range, a continuous high-value band appears from ROI2 to ROI4, with the most pronounced response around $Re_{\Gamma }\approx 533$. This indicates stronger local renewal in the middle tube rows under this condition. At higher $Re_{\Gamma }$, $I_{R}$ remains relatively high, but the inter-row differences become weaker. Thus, $I_{R}$ is useful for identifying the tube rows with stronger local renewal and reflects the spatial distribution of local activity intensity within the tube bundle.

Figure 9c shows that $R_{HF}$ has a more selective spatial distribution. Unlike the continuous high-value band observed for $I_{R}$, elevated $R_{HF}$ values are mainly concentrated at ROI2 and occur primarily in the intermediate $Re_{\Gamma }$ range, especially around $Re_{\Gamma }\approx 533–627$, whereas most other rows remain at lower levels. This indicates that the high-frequency components are spatially localized within the tube bundle. Therefore, $R_{HF}$ is more suitable for identifying the tube rows where high-frequency perturbations are concentrated, and it reflects spatial differences in frequency composition.

In Figure 9d, $F_{B}$ further indicates that local renewal on different tube rows does not always occur in a continuous manner. At low to intermediate $Re_{\Gamma }$, several local high-value regions appear from ROI3 to ROI5, especially at ROI4, suggesting that renewal at these positions is more associated with burst-like strong events. At higher $Re_{\Gamma }$, high $F_{B}$ values become more scattered, and a continuous high-value band is no longer evident. Thus, $F_{B}$ does not simply indicate which row is more active overall, but rather identifies the tube rows where renewal is more intermittent and event-dominated.

These results indicate that a single descriptor is insufficient to fully characterize the local-state of different tube rows. $I_{R}$ describes the spatial distribution of renewal intensity, $R_{HF}$ reflects the concentration of high-frequency components, $F_{B}$ characterizes the intermittency of burst-like events, and $f_{p}$ provides complementary information on the dominant time scale. Combining these descriptors enables the local flow state to be characterized from four aspects: intensity, frequency composition, intermittency, and time scale. Therefore, the method can characterize not only the overall evolution with $Re_{\Gamma }$, but also the local state differences among tube rows within a smooth tube bundle, providing an interpretable basis for spatial comparison.

## 5. Method Validation and Parameter Robustness

All image sequences in this study were acquired after the flow rate, temperature, and optical conditions had stabilized, with each dataset corresponding to approximately 1 s of stable flow. The ROIs were fixed at the center of the main liquid-film region on each tube row and were used to characterize film activity in the main flow region, rather than the axially averaged behavior over the entire tube. Because the brightness of reflective images is jointly affected by interfacial morphology, reflection angle, illumination distribution, and camera noise, $M_{n}$, G, $I_{R}$, $R_{HF}$, $f_{p}$, and $F_{B}$ are all defined as image-derived proxy descriptors. To ensure that these proxies can be used for subsequent comparisons among operating conditions, this section examines the robustness and validity of the processing workflow in terms of the zero-response baseline, key parameter sensitivity, noise correction, and event-threshold definition.

### 5.1. Visual Diagnosis of Dynamic-Processing Parameters

**Figure 10:**
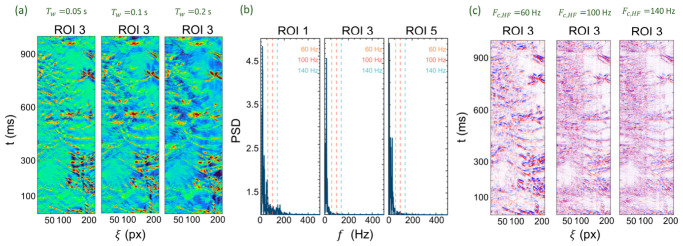
Visual diagnosis of key dynamic-processing parameters. (**a**) Influence of the detrending window $T_{w}$ on the normalized spatiotemporal map $M_{n}$ for ROI3. (**b**) Power spectral density distributions of the spatially averaged normalized activity signal for ROI1, ROI3, and ROI5; the dashed vertical lines indicate candidate high-frequency cutoffs $f_{c,HF}=60,\;100,\;140\;Hz$. (**c**) High-frequency spatiotemporal structures extracted using different $f_{c,HF}$ values for ROI3. In (**a**), colors indicate the normalized spatiotemporal response $M_{n}$; in (**c**), red and blue indicate positive and negative high-frequency components, respectively.

As shown in Figure 10, before the quantitative sensitivity analysis, the key dynamic-processing parameters are first examined visually from two perspectives: spatiotemporal patterns and spectral distributions. This figure mainly illustrates the processing roles of the detrending window $T_{w}$ and the high-frequency cutoff $f_{c,HF}$, and provides visual support for selecting their default values.

Figure 10a compares the normalized spatiotemporal maps $M_{n}$ of ROI3 under different detrending windows $T_{w}$. As $T_{w}$ increases from 0.05 s to 0.20 s, the dominant oblique streaks, localized high-activity regions, and overall texture distribution remain largely consistent. This indicates that the detrending window does not substantially alter the main apparent interfacial activity structures within this range. A smaller $T_{w}$ removes more slowly varying background components, making local rapid variations more prominent, but it may also attenuate some genuine low-frequency fluctuations. Conversely, a larger $T_{w}$ retains more low-frequency variations, but may also preserve slow illumination drift or quasi-static reflective background. Considering both the suppression of slowly varying background and the preservation of effective fluctuation structures, $T_{w}=0.1\;s$ is adopted as the default detrending window.

Figure 10b presents the power spectral density distributions of ROI1, ROI3, and ROI5, with the candidate high-frequency cutoffs $f_{c,HF}=60,\;100,\;140\;Hz$ marked. The spectral energy is mainly concentrated in the low-frequency range, whereas 60–140 Hz lies in the decay region after the dominant low-frequency fluctuations. If $f_{c,HF}$ is set too low, part of the dominant fluctuation energy will be included in the high-frequency component, so that $R_{HF}$ no longer specifically represents rapid perturbations. If $f_{c,HF}$ is set too high, the high-frequency integral becomes more sensitive to sharp reflective pulses and random noise, thereby reducing its ability to characterize valid apparent activity structures. Based on these spectral distributions, $f_{c,HF}=100\;Hz$ is adopted as the default cutoff frequency for separating dominant fluctuations from rapid perturbations.

Figure 10c further shows the high-frequency spatiotemporal structures extracted under different $f_{c,HF}$. As $f_{c,HF}$ increases from 60 Hz to 140 Hz, the retained structures gradually change from relatively broad mid- to high-frequency streaks to finer and more fragmented rapid perturbations. At $f_{c,HF}=60\;Hz$, the high-frequency map still contains many mid-frequency fluctuation structures, indicating that the cutoff frequency is relatively low. At $f_{c,HF}=140\;Hz$, the retained structures are closer to localized fine perturbations and sharp responses, which may remove part of the valid apparent activity. In comparison, $f_{c,HF}=100\;Hz$ provides a reasonable balance between retaining rapid apparent activity and avoiding excessive inclusion of noise responses.

Therefore, the default values $T_{w}=0.10\;s$ and $f_{c,HF}=100\;Hz$ fall within a range with clear image-domain interpretation. The subsequent quantitative sensitivity analysis further examines the influence of these parameters on descriptor magnitudes and ROI spatial ranking.

### 5.2. Zero-Response Baseline and Structural Artifact Check

To test whether the processing workflow generates structured artifacts in the absence of actual liquid-film motion, static calibration-plate images were used as zero-response inputs. The calibration-plate images were acquired using the same camera position, illumination arrangement, exposure parameters, sampling frequency, and ROI definitions as those used in the falling-film experiments. They were then processed using the same workflow as the real flow images, including background construction, robust normalization, dynamic renewal field G calculation, spectral analysis, and event detection. Since no liquid-film fluctuations, liquid-column impingement, local breakup, rewetting, or propagation processes occur on the calibration plate, the processed results should ideally not exhibit directional, continuous, or locally organized spatiotemporal structures.

**Figure 11:**
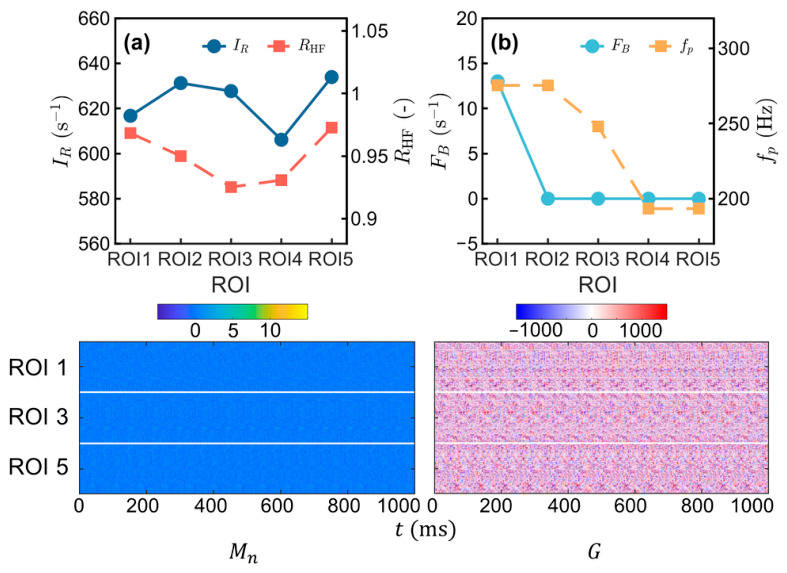
Zero-response baseline test using static calibration-board images. (**a**) Zero-response values of $I_{R}$ and $R_{HF}$ for different ROIs. (**b**) Zero-response values of $F_{B}$ and $f_{p}$ for different ROIs. The lower panels show the corresponding $M_{n}$ and G maps for selected ROIs under static input. The absence of organized streaks, localized clusters, or directional structures indicates that the processing workflow does not generate structured pseudo-waves in the zero-response condition.

Figure 11 shows the $M_{n}$ and G spatiotemporal maps under the zero-response input. The $M_{n}$ maps mainly show low-amplitude random textures, and no oblique streaks, localized high-response clusters, or continuous propagation structures typical of real falling-film images appear in any ROI. This indicates that background subtraction and MAD-based normalization do not amplify weak noise in static images into patterns resembling film fluctuations. Although some random red and blue spots are visible in the G maps, these arise from the amplification of camera noise, slight illumination perturbations, quantization errors, and background residuals by temporal differencing. These spots do not form stable alternating positive–negative bands, propagation directions, or localized burst regions, and should therefore be interpreted as noise responses in the dynamic renewal field rather than apparent interfacial renewal events.

Under the zero-response condition, $I_{R}$ is not strictly zero, mainly because residual noise is amplified by temporal differencing. Therefore, this value is treated as the $I_{R}$ noise floor for the current optical system and processing workflow, rather than being artificially set to zero. $F_{B}$ is zero in most ROIs, with only a few random threshold-crossing events appearing in individual ROIs. This indicates that the event-detection criterion does not generally produce spurious burst events under static input. $R_{HF}$ may be relatively high under the zero-response condition, but this occurs because the static images lack genuine low- or mid-frequency apparent interfacial structures, and the remaining signal is dominated by random high-frequency noise. It therefore does not indicate the presence of real high-frequency liquid-film fluctuations. Similarly, $f_{p}$ is only a formal peak in the PSD of a finite-length random signal and has no physical meaning when clear spatiotemporal structures and stable spectral peaks are absent.

Therefore, the descriptors obtained from real flow conditions are used for state comparison only when the following conditions are met simultaneously: first, the scalar response is clearly higher than the zero-response baseline; second, identifiable streaks, clusters, localized renewal, or propagation structures are present in the $M_{n}$ or G maps; and third, the corresponding trends remain stable in parameter-sensitivity and repeatability tests. The zero-response baseline test indicates that the processing workflow does not generate structured false fluctuations under static input, and it provides reference criteria for determining which descriptors can be assigned flow-related physical interpretation.

### 5.3. Sensitivity Analysis of Data-Construction Parameters

**Figure 12:**
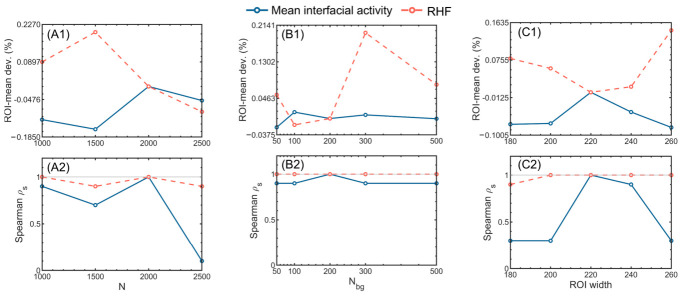
Sensitivity of visual-proxy descriptors to data-construction parameters. (**A1**,**A2**) Effect of the image sequence length N. (**B1**,**B2**) Effect of the number of background frames $N_{bg}$. (**C1**,**C2**) Effect of the ROI width $W_{ROI}$. The upper row shows the ROI-mean deviation of the descriptor magnitude relative to the default-parameter result, whereas the lower row shows the Spearman rank correlation coefficient $\rho_{s}$, which evaluates the consistency of ROI ordering under parameter variation. Blue curves denote the mean apparent activity descriptor, and red curves denote the high-frequency fraction $R_{HF}$.

After the zero-response baseline test, the sensitivity of the data-construction parameters was examined. As shown in Figure 12, the image sequence length N, number of background frames $N_{bg}$, and ROI width $W_{ROI}$ were varied to evaluate their influence on the descriptor outputs. For each parameter, the ROI-mean deviation and Spearman rank correlation coefficient $\rho_{s}$ were used as evaluation metrics. The ROI-mean deviation quantifies the change in descriptor magnitude relative to the default-parameter result, whereas $\rho_{s}$ evaluates whether the relative ordering among ROIs remains stable. In the figure, the blue curves represent the mean apparent activity intensity, and the red curves represent the high-frequency fraction $R_{HF}$.

Figure 12 shows that, within the tested ranges, N, $N_{bg}$, and $W_{ROI}$ have only limited effects on the overall descriptor magnitude. The ROI-mean deviations of both the mean apparent activity intensity and $R_{HF}$ remain low, indicating that these data-construction parameters do not substantially alter the overall statistical results.

**Figure 13:**
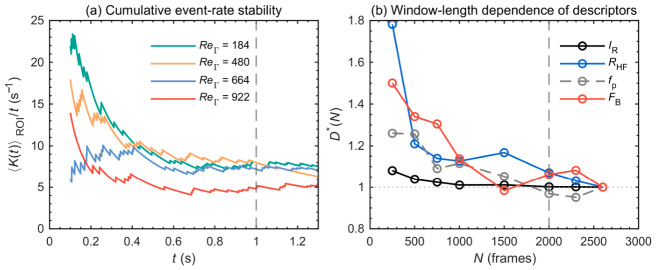
Observation-window sensitivity of burst-event statistics and image-derived descriptors. The vertical dashed line indicates the default 1 s observation-window length used in this study.

For the image sequence length N, the results show that 1000 frames or more are sufficient to obtain relatively stable overall statistics. However, an overly short sequence may contain only a few local events, such as liquid-column impingement, merging, or rewetting, especially at low Reynolds numbers or under strongly intermittent conditions. In such cases, the statistics may be affected by sporadic events. Conversely, a longer sequence increases the sample size but also increases computational cost and may include slow drift or local non-stationary variations during the experiment. In this study, N=2000 was adopted, corresponding to approximately 1 s of recording at 2000 fps. This duration covers multiple apparent renewal and fluctuation events while avoiding an excessively long time window, and its adequacy was further examined by an observation-window check. The observation-window sensitivity results are shown in Figure 13.

To further justify this choice for intermittent falling-film statistics, an additional observation-window check was performed using 2600-frame image sequences under representative $Re_{\Gamma }$ conditions. The cumulative burst-event rate $F_{B}$, averaged over the five ROIs, was calculated as a function of N. Short observation windows showed larger fluctuations because only a limited number of intermittent renewal events were sampled. As N increased, the cumulative event-rate curves gradually became flatter, and the variation near N=2000 was much smaller than that in the initial stage.

(37) $$D_{c,r}^{*}\left(N\right)=\frac{D_{c,r}\left(N\right)}{D_{c,r}\left(2600\right)}$$

where D denotes $I_{R}$, $R_{HF}$, $f_{p}$, or $F_{B}$, c denotes the operating condition, r denotes the ROI index, and N is the number of frames used in the analysis. The results show that short windows can produce noticeable deviations, especially for $R_{HF}$, $f_{p}$, and $F_{B}$, whereas $I_{R}$ is less sensitive over the tested range. As N increases, the descriptor values generally approach the 2600-frame reference values and become relatively stable near N=2000. Therefore, the 2000-frame window provides a practical compromise between descriptor stability and computational cost. This stability refers to the robustness of the image-derived optical descriptors with respect to observation-window length, rather than convergence of true physical interfacial quantities.

The number of background frames $N_{bg}$ is used to construct the static background and reduce the influence of tube-wall texture, fixed reflections, and non-flow structures on the apparent activity field. If $N_{bg}$ is too small, the background image may be affected by a few instantaneous fluctuations or local highlights, causing some dynamic structures to be incorporated into the background. If $N_{bg}$ is too large, the computational burden increases with limited improvement. The sensitivity results show that, within $N_{bg}=50–500$, the mean values and ROI ordering of the main descriptors remain stable. This indicates that the temporal-median background is robust to changes in the number of background frames. Therefore, $N_{bg}=200$ was adopted.

The ROI width $W_{ROI}$ determines the range of local spatial averaging. If the ROI is too narrow, the results may be overly affected by local highlights, isolated liquid columns, or individual ripple structures, and may not represent the overall state of the main flow region on that tube row. If the ROI is too wide, it may include the edge of the main flow region, weakly wetted zones, or other non-target areas, mixing responses from different spatial regions. The sensitivity results show that $W_{ROI}$ has little influence on the overall mean, but it can affect the ROI ordering of the mean apparent activity intensity at some widths. Thus, ROI width has limited influence on the overall magnitude but may affect the interpretation of subtle inter-row differences. In this study, $W_{ROI}=220$ px was adopted because it lies near the middle of the tested range and provides stable ROI ordering.

Therefore, the default values N=2000, $N_{bg}=200$, and $W_{ROI}=220$ px were used in the subsequent analysis.

### 5.4. Sensitivity Analysis of Dynamic-Processing Parameters

Dynamic-processing parameters affect the calculation of $M_{n}$, G, and their derived descriptors. This section examines the sensitivity of the detrending window $T_{w}$, the high-frequency cutoff $f_{c,HF}$, and the pre-differentiation low-pass cutoff $f_{c,LP}$. These parameters control slow-background removal, the spectral separation between dominant fluctuations and rapid perturbations, and the dynamic frequency range retained before temporal differencing, respectively.

**Figure 14:**
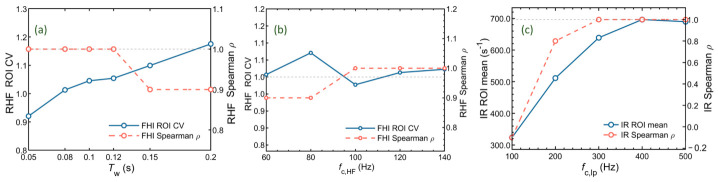
Sensitivity of visual-proxy descriptors to dynamic-processing parameters. (**a**) Effect of the detrending window $T_{w}$ on the high-frequency descriptor and its ROI-ranking consistency. (**b**) Effect of the high-frequency cutoff $f_{c,HF}$ on the high-frequency descriptor and its ROI-ranking consistency. (**c**) Effect of the pre-differentiation low-pass cutoff $f_{c,LP}$ on the apparent renewal intensity $I_{R}$ and its ROI-ranking consistency. The Spearman coefficient $\rho_{s}$ is used to evaluate whether the relative ordering among ROIs is preserved when the processing parameters are varied. The gray dashed horizontal line indicates a Spearman rank correlation coefficient of 1.

As shown in Figure 14, the main $M_{n}$ streak structures and localized high-activity regions are preserved when $T_{w}$ varies within the tested range. The spectra also show that the dominant energy is concentrated at low frequencies, while 60–140 Hz lies in the decay region after the main fluctuation band. The extracted high-frequency structures gradually change from broader mid-frequency streaks to finer fragmented perturbations as $f_{c,HF}$ increases.

For $T_{w}$, a short window may subtract part of the effective low-frequency activity as background, whereas a long window may retain slow illumination drift or quasi-static reflections. The results show that the ROI ordering remains stable for $T_{w}=0.05–0.12\;s$, while $\rho_{s}$ decreases when $T_{w}\geq 0.15\;s$. Therefore, $T_{w}=0.10\;s$ is adopted as the default value, balancing slow-background suppression and preservation of the main fluctuation structures.

For $f_{c,HF}$, a low cutoff would include part of the dominant fluctuation energy in the high-frequency fraction, whereas a high cutoff would make $R_{HF}$ more sensitive to sharp reflective pulses and noise. The ROI ordering remains stable when $f_{c,HF}\geq 100\;Hz$, and the PSD results indicate that 100 Hz lies after the dominant low-frequency band. Thus, $f_{c,HF}=100\;Hz$ is used as the default cutoff for separating dominant fluctuations from rapid perturbations.

For $f_{c,LP}$, the effect is mainly reflected in G and $I_{R}$. Since temporal differencing amplifies high-frequency components, an overly low $f_{c,LP}$ would remove valid rapid apparent renewal, whereas an overly high value would introduce more camera noise, illumination perturbations, and random reflective fluctuations into the differencing step. The results show that $I_{R}$ increases with $f_{c,LP}$ and reaches a plateau at approximately 300 Hz; the ROI ordering also becomes stable when $f_{c,LP}\geq 300\;Hz$. Therefore, $f_{c,LP}=300\;Hz$ is adopted as the default value.

Based on the spatiotemporal structures, spectral distributions, and ROI-ordering stability, the default dynamic-processing parameters are set as $T_{w}=0.10\;s,\;f_{c,HF}=100\;Hz,\;f_{c,LP}=300\;Hz$.

### 5.5. Noise-Band Starting Frequency and Its Effect on $I_{R}$ Correction

In the calculation of the apparent renewal intensity $I_{R}$, temporal differencing amplifies high-frequency components in the normalized fluctuation field $M_{n}$. Therefore, $I_{R}$ may contain both flow-related rapid grayscale variations and non-flow contributions, such as camera noise, slight illumination fluctuations, and quantization errors. A high-frequency noise band is therefore used to estimate the noise contribution $I_{R,noise,i}$, and the noise-corrected apparent renewal intensity is calculated as

(38) $$I_{R,i}\equiv I_{R,corr,i}=\sqrt{\max\left[I_{R,meas,i}^{2}-I_{R,noise,i}^{2},0\right]}$$

Here, $f_{n,start}$ defines the starting frequency of the high-frequency noise band. This parameter determines which frequency range is treated as dominated by random noise or unstructured optical disturbances, rather than by flow-related apparent responses such as column-to-sheet transition, liquid merging, local breakup, rewetting, or reflective-structure reorganization.

**Figure 15:**
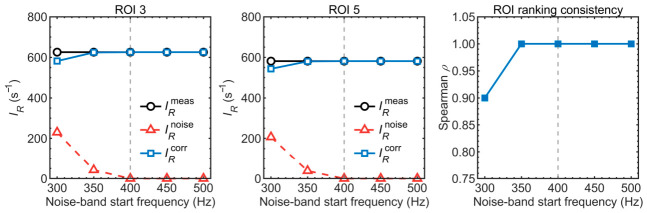
Effect of noise-band start frequency on the correction of apparent renewal intensity. The left and middle panels show the measured apparent renewal intensity $I_{R,meas}$, the estimated noise contribution $I_{R,noise}$, and the corrected apparent renewal intensity $I_{R}$ for ROI3 and ROI5, respectively. The right panel shows the Spearman rank correlation coefficient $\rho_{s}$ of the ROI ordering as the noise-band start frequency $f_{n,start}$ varies. The vertical dashed line indicates the default noise-band starting frequency of 400 Hz used for the noise-correction calculation.

If $f_{n,start}$ is too low, the noise band may extend into the mid- to high-frequency range that still contains valid apparent activity. In this case, $I_{R,noise,i}$ is overestimated, leading to excessive subtraction in $I_{R,i}$. When $f_{n,start}=300\;Hz$, the estimated noise contributions in ROI3 and ROI5 increase noticeably, and the corrected $I_{R}$ decreases by approximately 7% relative to the default result. This suggests that part of the valid rapid apparent renewal response may have been included in the noise estimate. The effect of the noise-band starting frequency on IR correction and ROI-ordering consistency is shown in Figure 15.

Conversely, if $f_{n,start}$ is too high, the noise estimate relies only on the extreme high-frequency tail. This avoids removing flow-related responses, but narrows the frequency band available for noise-floor estimation and may underestimate the noise contribution. In the present dataset, when $f_{n,start}$ increases to 350 Hz, $I_{R,noise}$ decreases rapidly and $I_{R,corr}$ approaches the default result. Further increasing $f_{n,start}$ to 400–500 Hz leads to a stable plateau, with $I_{R,corr}$ approaching $I_{R,meas}$. The ROI-averaged $I_{R,corr}$ is approximately $582.7\;s^{-1}$ at 300 Hz and stabilizes at about $627.7\;s^{-1}$ over 400–500 Hz. This indicates that components above approximately 350 Hz are mainly associated with unstructured high-frequency perturbations, and the risk of over-subtracting valid apparent activity is reduced.

The ROI-ordering results further show that $f_{n,start}$ mainly affects the magnitude of noise subtraction rather than the spatial distribution trend. Except for a slight decrease in the Spearman rank correlation coefficient at 300 Hz, the ordering among the five ROIs remains stable for $f_{n,start}\geq 350\;Hz$. Thus, reasonable variation in $f_{n,start}$ does not change the main judgment of which tube row exhibits stronger apparent renewal; it mainly affects how conservatively the high-frequency noise contribution is subtracted.

Based on these results, $f_{n,start}=400\;Hz$ is adopted as the default noise-band starting frequency. This value lies in the stable plateau region of both $I_{R,corr}$ and ROI ordering, while remaining sufficiently separated from the main flow-related activity band. In all subsequent comparisons, the same $f_{n,start}=400$ is used for different Re_Γ values and tube-row positions to maintain a consistent noise-correction baseline.

### 5.6. Influence of the Event-Identification Threshold on $F_{B}$

Unlike $I_{R}$ and $R_{HF}$, the burst-event rate $F_{B}$ is a threshold-based count. Therefore, its value depends directly on the event-identification threshold $k_{\sigma }$. For each ROI, event detection is performed on the spatially averaged dynamic renewal signal ${\bar{G}}_{i}(t)$, with the threshold defined as

(39) $$\Theta_{B,i}=k_{\sigma }\sigma_{\bar{G},i}$$

where $\sigma_{\bar{G},i}$ is the standard deviation of ${\bar{G}}_{i}(t)$. A strong apparent renewal event is identified when $|{\bar{G}}_{i}(t)|>\Theta_{B,i}$, and only the rising edge of each continuous supra-threshold interval is counted as one independent event.

**Figure 16:**
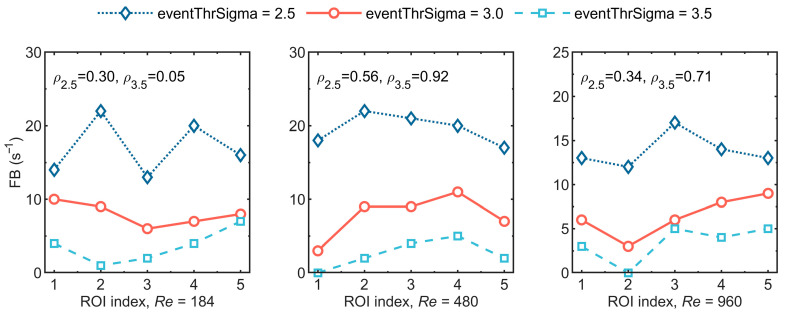
Effect of event-detection threshold on burst-event rate. The three panels correspond to representative Reynolds numbers: $Re_{\Gamma }=184,\;480\;and\;960$. In each panel, $F_{B}$ is plotted against ROI index for three threshold coefficients, $k_{\sigma }=2.5,\;3.0\;and\;3.5$. The values of $\rho_{s}$ reported in each panel indicate the Spearman rank correlation between the ROI ordering obtained with $k_{\sigma }=2.5$ or 3.5 and that obtained with the default threshold $k_{\sigma }=3.0$.

The threshold $k_{\sigma }$ controls the balance between detecting burst-like renewal events and excluding weak background fluctuations. A low threshold may count ordinary sheet-flow undulations, small reflective variations, or noise responses as events, whereas a high threshold may retain only a few extreme peaks and miss moderate but valid renewal processes. The sensitivity of FB to the event-identification threshold is shown in Figure 16.

Sensitivity tests were conducted at $Re_{\Gamma }=184,\;480\;and\;960$, representing column flow, the column-to-sheet transition regime, and sheet flow, respectively. As $k_{\sigma }$ increases from 2.5 to 3.5, the ROI-averaged $F_{B}$ decreases for all three cases. For $Re_{\Gamma }=184$, $F_{B}$ decreases from approximately 17.0 to 8.0 and $3.6\;s^{-1}$; for $Re_{\Gamma }=480$, from 19.6 to 7.8 and $2.6\;s^{-1}$; and for $Re_{\Gamma }=960$, from 13.8 to 6.4 and $3.4\;s^{-1}$. This confirms that the absolute value of $F_{B}$ should be interpreted as a threshold-dependent image-derived event rate, rather than a parameter-independent physical frequency.

At $k_{\sigma }=2.5$, the event count is high, but weak perturbations are also included. For the transition case at $Re_{\Gamma }=480$, the five ROIs give similar $F_{B}$ values of approximately $17–22\;s^{-1}$, indicating reduced discrimination of inter-row differences. At $k_{\sigma }=3.5$, only a small number of strong peaks are retained. Zero-event cases appear for ROI1 at $Re_{\Gamma }=480$ and ROI2 at $Re_{\Gamma }=960$, suggesting that the threshold is too strict for stable event statistics. The Spearman rank correlation also shows reduced ordering stability at $k_{\sigma }=2.5$, and at $k_{\sigma }=3.5$ for the low-$Re_{\Gamma }$ column-flow case.

Therefore, $k_{\sigma }=3.0$ is adopted as the default event-identification threshold. This value avoids excessive counting of weak fluctuations at $k_{\sigma }=2.5$, while reducing the risk of insufficient event samples and local zero counts at $k_{\sigma }=3.5$. It provides a consistent threshold criterion for comparing the relative occurrence frequency of burst-like apparent renewal events among different $Re_{\Gamma }$ values and tube-row positions.

## 6. Discussion on Methodological Positioning and Practical Applicability

### 6.1. Methodological Positioning and Contribution of the Image-Derived Descriptor Framework

The main contribution of this study is the construction and validation of an image-derived descriptor framework for reflective high-speed images. For falling-film flow over horizontal tube bundles, grayscale variations in the raw images are jointly affected by free-surface morphology, local reflection direction, highlight distribution, wetting-boundary migration, and camera and illumination conditions. Direct inference of film thickness, wave height, or interfacial velocity from grayscale intensity generally requires additional geometric and optical calibration. For this reason, the present study converts complex brightness variations recorded under a fixed optical configuration into reproducible optical proxies that can be compared across ROIs and operating conditions. These proxies are used to compare the intensity, spatiotemporal distribution, frequency composition, and intermittent events of interfacial activity.

As shown in Figure 4, the proposed method includes background modeling, robust normalization, temporal-gradient construction, spectral analysis, noise correction, and event detection. These steps are established image- and signal-processing tools, while the contribution of this study lies in their integrated use and targeted validation for reflective high-speed falling-film images. Specifically, the method establishes a standardized conversion path from reflective high-speed images to optical proxies and constructs a multilevel descriptor system consisting of $M_{n}$, G, $I_{R}$, $R_{HF}$, $f_{p}$, and $F_{B}$. Zero-response testing, noise-correction testing, and parameter-sensitivity analysis are used to clarify the reliability and interpretation limits of the framework for comparative monitoring under fixed optical conditions. The parameter-sensitivity analysis also examines whether the main descriptor trends and the ROI condition rankings are preserved, including $\rho_{s}$-based rank-order consistency where applicable.

The use of kymograph-like spatiotemporal representation and PSD analysis also follows this descriptor-oriented framework. Conventional STIV or kymograph analyses commonly infer propagation direction or apparent velocity from texture inclination in spatiotemporal maps, while standalone spectral diagnostics mainly focus on the temporal energy distribution at selected pixels or ROIs. In reflective liquid-film images, bright spots and dark regions may arise from instantaneous changes in interfacial curvature, surface slope, wetting state, and specular reflection conditions. Directly interpreting texture slopes as interfacial velocities can therefore introduce physical ambiguity. In this study, the kymograph-like representation is used to organize and display the normalized optical fluctuation field $M_{n}$ and the normalized dynamic renewal field G, and PSD analysis is incorporated into the descriptor system to characterize the time scales and frequency composition of the dynamic optical signals. The focus of this study is thus the construction of a reproducible descriptor-generation and validation workflow, rather than a simple combination of kymograph visualization and PSD analysis.

On this basis, the corrected apparent interfacial renewal intensity $I_{R}$, high-frequency optical-fluctuation fraction $R_{HF}$, dominant response frequency $f_{p}$, and burst-event rate $F_{B}$ characterize the image signal in terms of overall dynamic activity, short-time-scale fluctuation contribution, dominant time scale, and intermittency of strong optical events, respectively. Compared with optical-flow methods, cross-correlation methods, frequency–wavenumber analysis, POD, and DMD, the proposed framework places greater emphasis on region-resolved state description, ROI ranking, and operating-condition discrimination than on local velocity-field reconstruction or dominant-mode extraction. This design is more suitable for comparative engineering monitoring under fixed camera and illumination conditions. These descriptors should be understood as comparative diagnostic quantities reflecting interfacial optical activity and should not be directly interpreted as measurements of film thickness, interfacial velocity, or heat-transfer coefficient.

The computational cost of the proposed framework mainly arises from background subtraction, spatial averaging, ROI-based normalization, and FFT/PSD-based spectral analysis. The workflow consists primarily of linear image operations and fast Fourier transforms, without iterative inversion, model training, or full-field optical-flow estimation. Thus, the computational burden is relatively low and scales mainly with the number of frames, ROIs, and ROI size. The MATLAB implementation used here (MATLAB R2024a, MathWorks, Natick, MA, USA) was designed for offline batch analysis; however, when ROI positions, the background model, and processing parameters are fixed in advance, the core descriptor extraction can be adapted to online or near-real-time processing using streaming image input and sliding-window spectral updates. Strict real-time deployment would still depend on camera frame rate, image-transfer bandwidth, and processing hardware.

### 6.2. Physical Meaning and Engineering Significance of the Image-Derived Descriptors

Under reflective imaging conditions, the liquid-film free surface can be regarded as an unsteady reflective surface that evolves continuously with the flow. Local film thickness, interfacial curvature, surface slope, migration of the wetting boundary, liquid-column impact, liquid-bridge formation and rupture, wave passage, and the spreading or breakup of sheet-like films can all alter the local reflection direction and highlight distribution, producing brightness variations in the images. Under fixed illumination, viewing angle, exposure, and ROI settings, the grayscale fluctuations obtained after background subtraction and robust normalization can be used as the optical response of interfacial deformation and wetting-state evolution. The main optical meanings, practical uses, and interpretation boundaries of the descriptors are summarized in Table 5. These descriptors characterize interfacial activity in the same image sequence from the perspectives of spatial distribution, temporal gradient, frequency composition, and event occurrence, and can therefore be used together to compare local flow states at different tube-row positions and under different $Re_{\Gamma }$ conditions.

Synchronized physical measurements were outside the scope of this study, but the proposed descriptors can still be related qualitatively to conventional falling-film diagnostics. The normalized optical fluctuation field $M_{n}$ reflects changes in apparent wetting morphology, interfacial deformation, and wetting-boundary migration, quantities often examined through film-thickness, wetted-area, or interface-tracking measurements. The dynamic renewal field G and the corrected apparent interfacial renewal intensity $I_{R}$ capture rapid optical changes associated with wave passage, liquid-column impact, local rewetting, and liquid-bridge formation or rupture. In future calibrated studies, these descriptors could be compared with renewal-related thickness, wetted-area, or thermal-response measurements.

The high-frequency fraction $R_{HF}$, dominant response frequency $f_{p}$, and burst-event rate $F_{B}$ describe short-time-scale fluctuations, dominant optical time scales, and intermittent strong events. They are conceptually related to wave-frequency spectra and visually counted interfacial events. In the present study, these links are used only to support physical interpretation and comparative diagnosis. Quantitative conversion to film thickness, interfacial velocity, wetted-area ratio, or heat-transfer coefficient would require synchronized calibration using independent physical or thermal measurements.

As $Re_{\Gamma }$ increases, the variations in the descriptors are consistent with the observed flow-regime evolution. At low $Re_{\Gamma }$, the liquid is mainly transferred in the form of discrete liquid columns. The wetting coverage is relatively sparse, and the optical activity is concentrated near the impingement sites. Accordingly, $I_{R}$ and $R_{HF}$ are usually at relatively low levels. In the transition from column flow to sheet-like flow, reorganization processes such as lateral spreading and merging of liquid columns, formation and rupture of liquid bridges, local impact, and rewetting become more frequent. The interfacial morphology changes more rapidly, and G, $I_{R}$, $R_{HF}$, and $F_{B}$ tend to increase. At higher $Re_{\Gamma }$, the sheet-like coverage becomes more continuous, and the flow activity gradually shifts from localized strong transients to more spatially distributed fluctuations. Discrete strong-renewal events decrease, and $R_{HF}$ and $F_{B}$ may reach a plateau or decrease. These interpretations correspond to the observations obtained under the tube-bundle structure and optical configuration used in this study. Under different tube diameters, tube spacings, working fluids, or illumination conditions, the absolute magnitudes and local details of the descriptors may change.

These descriptors provide a non-contact way to characterize interfacial activity between tube rows over a large field of view. They are useful for comparing flow states under different $Re_{\Gamma }$ conditions, locating active wetting or renewal regions, and providing image-based evidence for flow-regime transition. In practical heat- and mass-transfer processes, the detailed flow behavior may differ from that under isothermal conditions because of temperature gradients, phase change, or concentration effects. However, the main falling-film flow patterns and the associated interfacial fluctuations, such as column impact, wave passage, liquid-bridge formation and rupture, intermittent rewetting, and sheet-film spreading or breakup, are expected to remain highly similar. These interfacial processes also produce reflective optical activity, which suggests that the proposed descriptors may be useful for comparative monitoring in thermal-process applications. The fixed-ROI strategy also limits the axial representativeness of the descriptors. This study does not assume that a single ROI represents the complete liquid-film distribution over the 300 mm tube length. The reported $M_{n}$, G, and scalar descriptors are local image-derived optical proxies within the selected field of view, and should therefore be interpreted as local comparative diagnostic quantities rather than axial averages over the full tube span. Axial liquid maldistribution, end effects, and spatially varying wetting conditions may lead to different descriptor magnitudes at other axial positions. The fixed ROI was used to maintain consistent optical sampling and processing conditions among different $Re_{\Gamma }$ values and tube rows. For long tubes or systems with strong axial non-uniformity, the workflow can be extended by using multiple axial ROIs, multiple camera views, or scanning/stitching measurements.

The proposed method is mainly suited to comparative online monitoring under a fixed optical configuration. When it is applied to practical heat exchangers or other engineering devices, reproducible imaging conditions should first be established, including stable illumination, a fixed viewing angle, appropriate exposure, avoidance of image saturation, and consistent ROI definition. When the device structure, camera, lens, light source, working fluid, or tube-bundle geometry changes, spatial-scale calibration, flat-field or background calibration, zero-flow/static baseline testing, noise-band estimation, and event-threshold verification should be performed again to ensure descriptor comparability among different operating conditions. For state identification, trend monitoring, and ROI ranking within the same device, these optical and signal-processing calibrations can generally support the use of the descriptors as image-derived optical proxies. If quantitative relationships between the descriptors and film thickness, wetting ratio, interfacial velocity, or heat-transfer coefficient are required, independent physical or thermal measurements should be introduced for synchronized calibration, such as film-thickness, wetted-area, wall-temperature, heat-flux, or heat-transfer-coefficient measurements. It should be emphasized that the present experiments were conducted under isothermal conditions, and no synchronized measurements of wall temperature, heat flux, or heat-transfer coefficient were performed. Therefore, the proposed descriptors are used as image-derived optical proxies for interfacial activity, wetting morphology, renewal behavior, and intermittency, rather than as direct thermal quantities. Their relevance to heat-transfer applications arises from the fact that falling-film heat transfer is affected by interfacial processes such as wetting coverage, interfacial renewal, wave-induced mixing, and intermittent rewetting, all of which can also modify the reflective image response. Establishing quantitative correlations between these image-derived descriptors and heat-transfer performance will require future experiments with synchronized thermal diagnostics.

The proposed framework is transferable primarily as a processing workflow. The descriptor magnitudes are configuration-dependent optical proxies and are affected by illumination direction and stability, camera view, lens, exposure time, frame rate, spatial resolution, depth of field, and image saturation. Therefore, comparisons should be made under fixed and well-documented imaging conditions. If the optical setup is changed, spatial-scale calibration, flat-field or background calibration, zero-flow/static baseline testing, noise-band estimation, filtering-parameter selection, and event-threshold verification should be repeated.

Application to other working fluids or tube geometries also requires case-specific validation. Fluid transparency, refractive index, color, impurities, bubble or droplet entrainment, viscosity, surface tension, and wettability may alter the grayscale response. Tube diameter, tube pitch, bundle arrangement, surface condition, curvature, shadowing, and occlusion can change the reflection pattern and ROI visibility. In industrial operation, mechanical vibration, fouling, optical-window contamination, restricted optical access, high temperature or pressure, and time-varying illumination may further affect descriptor comparability. For these cases, renewed optical calibration and sensitivity checks are needed. Thus, the descriptors should be used as comparative image-derived optical proxies within a fixed or revalidated measurement configuration, rather than as direct measurements of film thickness, interfacial velocity, wetted area, or heat-transfer coefficient.

## 7. Conclusions

This study addresses the difficulty of directly interpreting high-speed reflective images of falling-film flow over horizontal tube bundles and establishes an image-based proxy-descriptor framework for state comparison. The method uses grayscale image sequences within fixed ROIs as input. Through temporal-median background subtraction, local spatiotemporal mapping, moving-average detrending, and MAD-based robust normalization, the normalized spatiotemporal map $M_{n}$ is obtained, from which the dynamic renewal field G is further constructed. The noise-corrected apparent renewal intensity $I_{R}$, high-frequency fraction $R_{HF}$, dominant frequency $f_{p}$, and burst-event rate $F_{B}$ are then extracted to characterize differences in local apparent interfacial activity among Reynolds numbers and tube-row positions. The main conclusions are as follows.

(1) The processed $M_{n}$ and *G* fields convert complex light–dark fluctuations in high-speed reflective images into comparable spatiotemporal responses. As the Reynolds number increases, the processed results capture the overall transition from discrete column-flow wetting toward more continuous sheet flow, while highlighting typical image features such as local streaks, activity clusters, and intermittent enhancement.
(2) The four scalar descriptors characterize local activity from complementary perspectives. $I_{R}$ reflects the temporal renewal intensity of apparent activity, $R_{HF}$ represents the proportion of rapid fluctuation components, $f_{p}$ indicates the dominant response time scale, and $F_{B}$ describes the occurrence frequency of strong burst-like renewal events. Their combined use is more suitable than a single descriptor for distinguishing column flow, the column-to-sheet transition regime, and sheet flow.
(3) The inter-row distributions show that local activity within the tube bundle does not vary synchronously among tube rows. $I_{R}$, $R_{HF}$, and $F_{B}$ reveal spatial differences in renewal intensity, high-frequency perturbations, and intermittent burst events, respectively. This indicates that the method can be used not only for overall regime comparison, but also for examining local non-uniformity within the tube bundle.
(4) The zero-response baseline, noise correction, and parameter-sensitivity analyses indicate that the processing workflow does not generate structured spurious fluctuations under static input. The main descriptors also retain stable regime differences and ROI rankings within the tested parameter ranges. Therefore, the proposed framework provides a non-contact visual sensing approach with a relatively low calibration burden for relative state assessment of falling-film flow over tube bundles.

## Figures and Tables

**Table 1:** Comparison of representative measurement and image-based methods for falling-film state characterization.

Method Category	Typical Output	Key Requirements	Main Constraints for Reflective Tube-Bundle Films	Role in This Study
Conductance/capacitance probes	Local film thickness or liquid presence	Electrical calibration; fixed probe position	Intrusive or semi-intrusive; pointwise; limited spatial coverage	Local reference measurement
PLIF/LIF-based optical diagnostics	Calibrated film-thickness or interface field	Optical calibration; dye/tracer; controlled illumination	Sensitive to curvature, reflection, refraction, occlusion, and optical access	Quantitative benchmark when calibration is available
Confocal chromatic or laser displacement sensing	Local or linewise film thickness/interface position	Geometric calibration; precise optical alignment	High local accuracy but limited coverage; difficult over multi-row curved bundles	Local validation or calibration reference
High-speed visualization	Image sequence of wetting morphology and interfacial motion	Stable illumination; fixed camera settings; sufficient contrast	Raw grayscale depends on curvature, highlights, shadows, and camera response	Raw sensing input for descriptor extraction
Optical flow/image cross-correlation	Apparent displacement or image-structure velocity	Trackable texture; brightness consistency; spatial calibration	Reflective patterns may change with slope, wetting boundary, or highlights	Motion-oriented reference method
STIV/kymograph analysis	Propagation direction, streak inclination, or apparent velocity	Coherent streaks; known time and length scales	Streak slopes may reflect moving highlights as well as material motion	Related spatiotemporal representation
Frequency–wavenumber/spectral analysis	Dominant frequency, wavenumber, spectral energy, or phase velocity	Adequate sampling; quasi-stationary wave field	Global spectra may miss local intermittent events and optical non-stationarity	$\mathrm{Spectral}\;\mathrm{basis}\;\mathrm{for}\;R_{HF}$ $\;\mathrm{and}\;f_{p}$
POD/DMD modal decomposition	Coherent modes, modal frequencies, temporal coefficients	Sufficient snapshots; preprocessing; interpretable modes	Modes can mix flow structures, illumination variation, and background response	Exploratory modal analysis
Proposed visual-proxy descriptor framework	$M_{n}$, G$,\;I_{R}$$,\;R_{HF}$$,\;f_{p}$$,\;\mathrm{and}\;F_{B}$ as image-derived optical proxies	Fixed optical setup; consistent ROI; defined processing parameters; baseline/noise checks	Provides comparative optical proxies, not absolute film thickness, velocity, or heat-transfer data	Low-calibration descriptor workflow for comparative state assessment

**Table 2:** Definitions and interpretations of the visual-proxy field quantities and scalar descriptors.

Quantity	Type	Interpretation
$M_{n,i}(t,s)$	Field quantity	Normalized apparent activity distribution
$G_{i}(t,s)$	Field quantity	Rapid renewal regions of the apparent activity pattern
$I_{R,i}$	Scalar descriptor	Apparent renewal intensity
$R_{HF,i}$	Scalar descriptor	Fraction of high-frequency rapid fluctuations
$f_{p,i}$	Scalar descriptor	Dominant response time scale
$F_{B,i}$	Scalar descriptor	Burst-like apparent renewal event rate

**Table 3:** Relative expanded uncertainty of bundle-averaged image-derived descriptors and the variance contribution propagated from $Re_{\Gamma }.$

Descriptor D	Median Relative $U_{95}(\bar{D})$ (%)	$C_{Re}$ Median [Max] (%)	Dominant Source
$I_{R}$	11.35	0.00 [2.00]	ROI-to-ROI variation
$R_{HF}$	24.05	0.00 [0.33]	ROI-to-ROI variation
$f_{p}$	29.63	0.00 [2.08]	ROI-to-ROI variation
$F_{B}$	25.95	0.00 [0.17]	image-related uncertainty

**Table 4:** Expert-based reference flow-regime criteria and auxiliary image-derived metrics.

Item	Definition or Criterion	Role in This Study
CF	The observed region is dominated by discrete liquid columns, with no evident sheet-like region.	Expert-based reference flow-state label
CSF	Neighboring liquid columns begin to merge, forming local sheet-like regions or initially connected liquid regions.	Expert-based reference flow-state label
PF	Sheet-like flow becomes more evident, while discontinuous coverage and residual columnar streams remain.	Expert-based reference flow-state label
FSF	The sheet-like liquid film covers most of the effective distribution width; $C_{s}\geq 0.90$ is used as an auxiliary criterion.	Expert-based reference flow-state label
$M_{n}$	Normalized spatiotemporal map of optical fluctuations.	Expert-based reference flow-state label
$A_{M}$	ROI-averaged normalized optical fluctuation intensity.	Used to check consistency with visual dynamic structures in the raw videos.
$P_{M}$	Fraction of spatiotemporal points satisfying	Characterizes the overall optical fluctuation level.
$C_{S}$	Ratio of the apparent sheet-coverage width $W_{s}$ to the effective distribution width $W_{eff}$.	Assists in evaluating sheet spreading and identifying FSF.
Transition interval	The $Re_{\Gamma }$ interval between two adjacent experimental conditions where a change in reference flow state occurs.	Represents the practical range of gradual flow-state transition.

**Table 5:** Physical interpretation and practical use of the image-derived descriptors.

Descriptor	Main Optical/Flow-Related Meaning	Interpretation
$M_{n}$	Normalized optical fluctuation field reflecting the spatiotemporal organization of local brightness variations caused by wetting morphology and interfacial deformation.	Identifies optically active regions and compares spatial response patterns; requires calibration before interpretation as film thickness.
G	Temporal-gradient field highlighting rapid changes in the normalized optical response.	Locates rapid renewal or reorganization regions; represents apparent optical renewal rather than a true interfacial renewal rate.
$I_{R}$	Noise-corrected overall intensity of apparent renewal within an ROI.	Compares renewal activity among ROIs and $Re_{\Gamma }$ conditions; should not be treated as a heat-transfer coefficient.
$R_{HF}$	Fraction of high-frequency optical fluctuations in the normalized activity signal.	Evaluates the relative contribution of short-time-scale perturbations; depends on the selected cutoff frequency.
$f_{p}$	Dominant response frequency of the optical fluctuation signal.	Provides an auxiliary time-scale indicator; may be ambiguous for broadband or multi-peak spectra.
$F_{B}$	Threshold-defined rate of strong burst-like apparent renewal events.	Quantifies intermittency of strong optical events; depends on the event threshold and is not a true breakup frequency.

## Data Availability

The original contributions presented in this study are included in the article. Further inquiries can be directed to the corresponding author.

## Abbreviations

The following abbreviations are used in this manuscript:

DMD	Dynamic mode decomposition
fps	Frames per second
LIF	Laser-induced fluorescence
MAD	Median absolute deviation
PLIF	Planar laser-induced fluorescence
POD	Proper orthogonal decomposition
PSD	Power spectral density
ROI	Region of interest
STIV	Space–time image velocimetry
